# Drug-free Immunotherapeutic Biomimetic Nanoparticles for Treating Triple-Negative Breast Cancer

**DOI:** 10.1021/acsnano.5c18774

**Published:** 2026-02-13

**Authors:** Ofri Vizenblit, Rawan Mhajne, Assaf Zinger

**Affiliations:** 1 Bioinspired Nano Engineering and Translational Therapeutics Lab, Department of Chemical Engineering, Technion−Israel Institute of Technology, Haifa 3200003, Israel; 2 Russell-Berrie Nanotechnology Institute, Technion − Israel Institute of Technology, Haifa 3200003, Israel; 3 Resnick Sustainability Center for Catalysis, Technion−Israel Institute of Technology, Haifa 3200003, Israel; 4 Bruce and Ruth Rappaport Cancer Research Center, Technion−Israel Institute of Technology, Haifa 3200003, Israel; 5 Cardiovascular Sciences Department, Houston Methodist Academic Institute, Houston, Texas 77030, United States; 6 Neurosurgery Department, Houston Methodist Academic Institute, Houston, Texas 77030, United States

**Keywords:** biomimicry, nanoparticles, immunotherapy, triple-negative breast cancer, tumor microenvironment

## Abstract

Tumor-associated macrophages (TAMs) are key drivers of tumor progression, metastasis, and immune evasion in triple-negative breast cancer (TNBC). Yet, most treatment strategies focus solely on tumor cells, neglecting the immunosuppressive tumor microenvironment (TME). Given the strong correlation between TAMs infiltration and poor prognosis, innovative therapeutic strategies that modulate TAMs dynamics are urgently needed. Here, we introduce MPsomes, macrophage biomimetic nanoparticles engineered to disrupt TAMs recruitment and alter the TME. Fabricated via a microfluidic approach, MPsomes integrate macrophage membrane proteins into lipid-based nanoparticles, retaining key surface markers essential for immune interactions. *In vitro*, MPsomes exhibited selective adhesion to inflamed endothelium, reducing macrophage recruitment in a flow chamber and Transwell migration assays. *In vivo*, systemic administration of MPsomes significantly reduced intratumoral TAMs populations and resulted in a pronounced inhibition of tumor growth compared to conventional liposomes. Notably, the therapeutic efficacy of MPsomes was comparable to that of FDA-approved anti-PD1 immunotherapy, further underscoring their potential as a drug-free, biomimetic alternative for TNBC treatment. These findings highlight the potential of MPsomes as a drug-free immunotherapeutic platform capable of reshaping the TME and inhibiting tumor progression, representing a previously unexplored therapeutic approach for TNBC.

## Graphical abstract

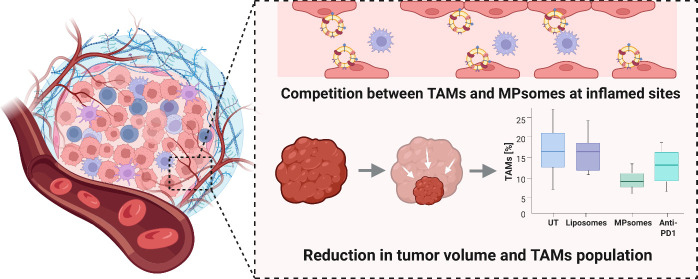

## Introduction

TNBC is the most aggressive breast cancer subtype, with the shortest survival and highest relapse rates.^1–3^ Despite significant increases in knowledge, TNBC treatment remains limited, with minimal impact on survival.^4^ Although most patients exhibit an increased risk of relapse, metastasis, and drug resistance within five years, initial studies were mainly focused on tumor cells, overlooking the tumor microenvironment (TME) signaling.^5,6^

As research expanded, it became clear that the TME plays a crucial role in TNBC progression and therapeutic resistance. Characteristics of the TME significantly influence disease progression, impacting tumor growth, cell invasion, metastasis, and the therapy response. Additionally, the TME comprises adaptive and innate immune cells such as T cells, dendritic cells, and macrophages. Among these, tumor-associated macrophages (TAMs) are the most abundant,^7^ derived from mononuclear cells and acting as tumor-promoting cells, are regulated by signals secreted within the TME. TAMs can change their phenotypes according to the signals from their environment.^7^ Recent studies have shown that higher TAMs infiltration in TNBC directly correlates with poor patient prognosis and reduced survival rates.^8,9^ Given the significant impact of TAMs on TNBC progression, researchers have developed macrophage-targeting therapeutic approaches. Based on these studies, TAMs-related therapeutic options have been developed, focusing on repolarizing TAMs,^9,10^ strengthening their antitumor activity,^9,11^ and inhibiting the recruitment of TAMs to the cancer sites using small-molecule inhibitors and antibodies.^12^ However, these broad-spectrum macrophage-targeting therapies are not specific and may cause systemic toxicities, as they indiscriminately affect all macrophages.

To address these challenges, we introduce MPsomes, macrophage-derived biomimetic lipid nanoparticles (NPs), as a novel, drug-free strategy to reprogram the TME. Specifically, we incorporated macrophage-derived membrane proteins, extracted from the murine macrophage cell line J774A.1 (ATCC, TIB-67), into synthetic lipid-based NPs to create macrophage-mimicking liposomes (i.e., MPsomes). This integration allowed the lipid bilayer to present surface proteins characteristic of macrophages, thereby conferring cell-mimetic properties onto the particles. By leveraging macrophage membrane proteins to outcompete native TAMs recruitment and inhibit their adhesion to inflamed endothelium, MPsomes offer a unique mechanism of action distinct from conventional nanotherapies.^13,14^ This biomimetic platform not only modulates immune cell dynamics but also circumvents the need for synthetic ligands or exogenous immunomodulators, reducing the risk of adverse immune activation.^9,15^ While several macrophage membrane-coated NPs have been reported, most rely on cloaking rigid inorganic or polymeric cores with intact cell membranes and are primarily designed as drug delivery vehicles.^16^ In contrast, MPsomes are liposome-based biomimetic nanoparticles engineered through controlled integration of macrophage-derived membrane proteins into a synthetic lipid bilayer rather than full membrane coating. This strategy preserves key macrophage-like surface functionalities while enabling precise control over composition, membrane fluidity, reproducibility, and scalability.^17^

Beyond immune modulation, inflamed endothelial cells also play a pivotal role in shaping the tumor microenvironment by promoting vascular inflammation and mediating the immune cell recruitment. Upon activation, these cells upregulate adhesion molecules and secrete chemokines that facilitate the trafficking of immune cells into the tumor.^18,19^ In TNBC, endothelial activation further supports tumor angiogenesis by releasing pro-angiogenic mediators, thereby sustaining tumor growth and immune evasion.^19^ This dual role of inflamed endothelial cells in immune cell recruitment and vascular remodeling underscores their importance in tumor progression and highlights them as attractive targets for NPs design.

Biomimetic NPs offer a promising avenue for overcoming the limitations of conventional NPs-based therapies by leveraging the natural properties of immune cells to enhance tumor targeting and modulate the TME.^17^ Biomimetic strategies represent a paradigm shift in the design of NPs, enabling next-generation platforms capable of effectively interfacing and interacting with complex biological systems through biomimicry of native cells.^20–24^ Examples of this include neuron mimicry using lipid-based NPs^25^ and platelet mimicry using Fe_3_O_4_ magnetic NPs coated with a platelet membrane.^26^ In particular, leukocyte-based biomimetic NPs demonstrate the potential for highly specific targeting of inflamed environments, such as TNBC tumors.^13,27,28^ This inflammation targeting is possible due to the presence of lymphocyte function-associated antigen 1 (LFA-1) on the leukocyte-based biomimetic NPs surfaces, which permits leukocytes to transiently bind to one of the molecules overexpressed in inflamed endothelia, intercellular adhesion molecule 1 (ICAM-1).^29^

Our results highlight the therapeutic potential of MPsomes as a biomimetic, drug-free strategy for TNBC treatment. We demonstrate that MPsomes exhibit a high association with inflamed endothelial cells while reducing macrophage recruitment *in vitro*. In an *in vivo* TNBC model, systemic MPsome administration markedly decreased the number of TAMs within the tumor, leading to significant tumor growth inhibition compared with liposome treatment. Notably, the therapeutic efficacy of MPsomes was comparable to that of the FDA-approved anti-PD1 immunotherapy, further validating their potential as a drug-free, biomimetic alternative capable of achieving immunomodulatory and antitumor effects on par with established checkpoint blockade therapies. Together, these findings underscore the promise of MPsomes as an efficient cancer-targeted nanoparticle platform and a compelling next-generation alternative to conventional therapeutic approaches.

## Results and Discussion

### 1 Reproducible Assembly and Characterization of Macrophage-Based Biomimetic NPs (MPsomes)

For the assembly and characterization of the macrophage-based biomimetic NPs (MPsomes), membrane proteins from a macrophage cell line (J774A.1, ATCC) were extracted and integrated into a liposomal nanoparticle lipid backbone using the microfluidic method described by Zinger.^13^ Next, we assessed the physical characteristics of both MPsomes and liposomes using dynamic light scattering (DLS) for the size distribution, polydispersity index (PDI), and zeta potential (ZP). Notably, the size and PDI of MPsomes and liposomes showed no significant difference on day 1. Specifically, the liposomes had an average size of 101.5 ± 15.1 nm and PDI of 0.16 ± 0.04, while the MPsomes had an average size of 100.9 ± 2.7 nm and PDI of 0.17 ± 0.02. Interestingly, MPsomes exhibited a statistically significantly more negative charge, approximately 2.2-fold greater than that of liposomes. Stability studies for 21 days in storage conditions (4 °C, PBS) revealed that MPsomes and liposomes maintained the same physicochemical properties as we assessed on day 1, with no significant changes in size, PDI, or ZP over the storage period (Figure 1A–C).

**Figure 1:**
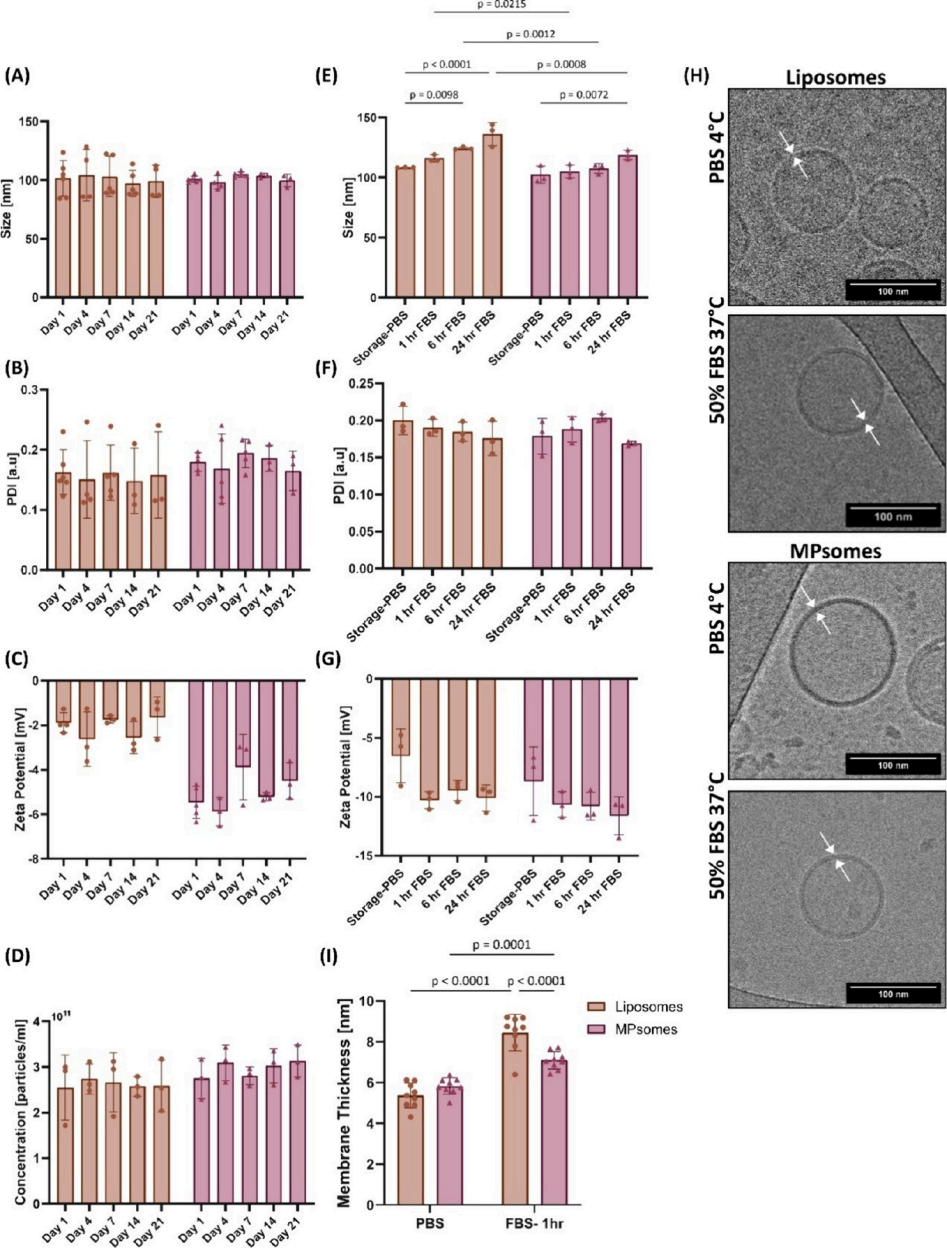
MPsomes and liposomes remain stable for up to 21 days when stored at 4 °C in PBS. However, their physicochemical properties change after incubation in serum at 37 °C. MPsomes and liposomes were assessed for their (A) size [nm], (B) polydispersity index (PDI) [a.u.], (C) zeta potential [mV], and (D) concentration [particles/mL] using Zetasizer Ultra Red for up to 21 days when stored at 4 °C in PBS. Moreover, both NPs groups were also assessed for their (E) size [nm], (F) PDI, and (G) zeta potential [mV] after incubation in FBS at 37 °C up to 24 h. (H) Cryo-TEM images of both NPs groups after 1 h of incubation in either storage conditions (4 °C, PBS) or serum conditions (37 °C, 50% FBS) illustrated that all groups maintained their spherical bilayer membrane structure, scale bar = 100 nm. (I) NPs’ lipid membrane thickness was measured in storage conditions (4 °C, PBS) and in serum at 37 °C. Results are shown as mean ± SD. Two-way ANOVA followed by uncorrected Fisher’s LSD test was used to determine statistical probabilities. *P* value ≤ 0.05 among means was considered statistically significant.

### 2 MPsomes Exhibit Changes in Their Pyrochemical Properties after Serum Incubation

Knowing MPsomes will be systemically injected to target inflamed endothelia in the TME, we assessed their serum stability vs liposomes by analyzing physicochemical properties after 1, 6, and 24 h in 50% FBS at 37 °C using DLS and cryo-TEM. Although both MPsomes and liposomes displayed significant changes in their physicochemical properties during serum incubation, these changes occurred earlier in the liposomes group compared to the MPsomes. The average diameter of liposomes increased by 1.2-fold after 6 h in 50% FBS compared to those stored in PBS. Notably, MPsomes exhibited a similar 1.2-fold increase in diameter only after 24 h in 50% FBS (Figure 1D–F). Notably, no significant alterations in PDI or ZP values were detected following serum incubation (Figure 1G).

To evaluate the changes in the NPs’ morphology and lipid bilayer thickness following serum incubation at 37 °C, we imaged the NPs groups using cryo-TEM and measured their membrane thickness. Cryo-TEM imaging revealed that MPsomes and liposomes maintained their round sphere morphology after 1 h of incubation in FBS. Next, we measured the membrane thickness of the NPs before and after the 1 h serum incubation (Figure 1H). Under storage conditions (4 °C in PBS), no significant difference in membrane thickness was observed between liposomes and MPsomes. However, after 1 h of incubation in 50% FBS at 37 °C, the membrane thickness of MPsomes increased by 1.2-fold compared to MPsomes in PBS, while the membrane thickness of liposomes showed a 1.6-fold increase compared to liposomes in PBS. Consequently, following the serum incubation, liposomes exhibited a thicker membrane than MPsomes (Figure 1I).

These findings suggest that while MPsomes and liposomes undergo structural changes during serum incubation, MPsomes demonstrate greater stability in their physicochemical properties over time.

### 3 Protein Profiling of Macrophage-Based Biomimetic NPs (MPsomes)

To characterize the biomimetic component of the MPsomes (i.e., macrophage-related membrane proteins), we conducted SDS-PAGE, complete proteomic analysis, and Western blot (WB) analysis of CD11b as a representative protein. First, the SDS-PAGE of MPsomes exhibited diverse protein bands with a consistent distribution across replicates, in contrast to liposomes, which showed no bands (Figure 2A).

**Figure 2:**
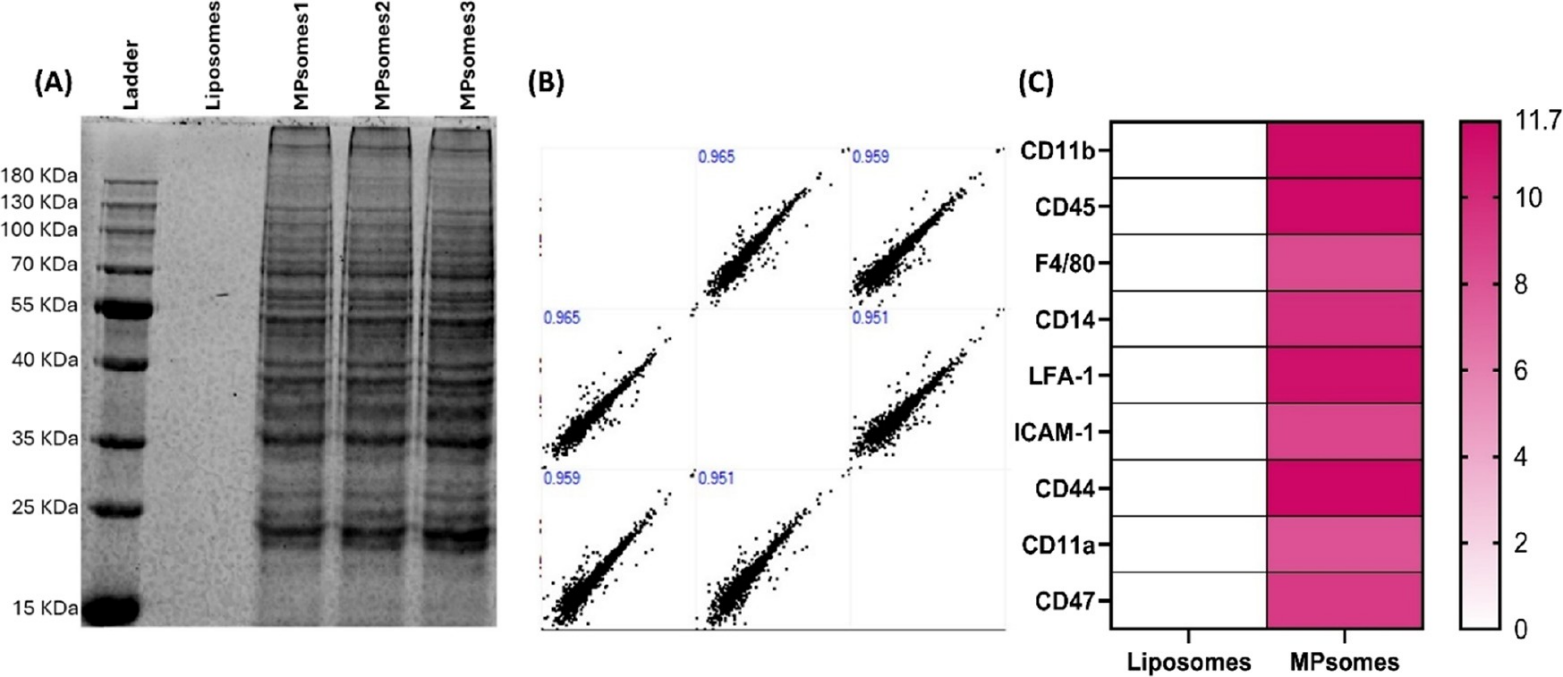
Protein profiling of MPsomes demonstrates reproducible assembly and the presence of macrophage-related membrane proteins. (A) Denaturing polyacrylamide gel electrophoresis followed by Coomassie Blue staining of three MPsomes replicates, compared to liposomes, was used to visualize the presence of macrophage protein integrated into MPsomes. (B) The Pearson correlation coefficient was calculated to assess the linear relationship between the protein intensities obtained from the full proteomic analysis of three MPsomes replicates and shows a significantly high correlation of the protein content, confirming reproducibility. (C) A heatmap representation of specific macrophage-related markers was obtained in the MPsome fractions compared to liposomes, as revealed in the proteomic analysis.

A comprehensive proteomic analysis of MPsomes revealed high reproducibility in protein intensities across three independent batches, as indicated by Pearson correlation analysis, showing a correlation coefficient greater than 0.9 (Figure 2B). Next, we evaluated the relative quantity of selected macrophage-related membrane proteins within the identified proteins from MPsomes (Figure 2C). As shown in Figure 2C, MPsomes contain key macrophage markers, including CD45, CD11b, and LFA-1, whereas liposomes do not contain any of these markers. Finally, to strengthen our findings, we analyzed the presence of CD11b, as it was presented in proteomic analysis as a leading marker in the MPsome fraction. Indeed, the WB analysis confirmed the presence of CD11b in MPsomes. Quantitative analysis of the band intensity in the WB showed that we enriched the CD11b protein concentration, as a representative marker for other macrophage membrane proteins in the extracted membrane protein fraction, by 1.25-fold (Figure S1).

### 4 MPsomes Did Not Increase Cell Toxicity *In Vitro*

Before evaluating the targeting ability of MPsomes and liposomes toward inflamed vein-endothelial *in vitro*, we assessed both NP’s toxicity levels. For this purpose, a 3-(4,5-dimethylthiazol-2-yl)-2,5-diphenyltetrazolium bromide (MTT) reduction assay to assess metabolic activity was performed on endothelial cells with several NPs concentrations. Briefly, murine vein-endothelial cells were incubated with increasing concentrations (0.1, 0.25, 0.5, and 1 mM) of NPs for 24 h, and then the MTT reagent was added. Viability percentages were calculated relative to untreated cells. Endothelial cells maintained 100% viability compared to untreated cells after 24 h of incubation with NPs at all tested concentrations (Figure 3A). To further ensure that MPsomes did not influence TAMs populations by directly eliminating macrophages, we examined their cytotoxicity on the J774 macrophage cell line in three activation states: nonactivated (Figure 3B), LPS-activated (pro-inflammatory, M1-like) (Figure 3C), and IL-4–activated (anti-inflammatory, M2-like)^30,31^ (Figure 3D). Across all NPs concentrations tested, no differences in viability were observed between MPsomes and liposomes in any activation state.

**Figure 3:**
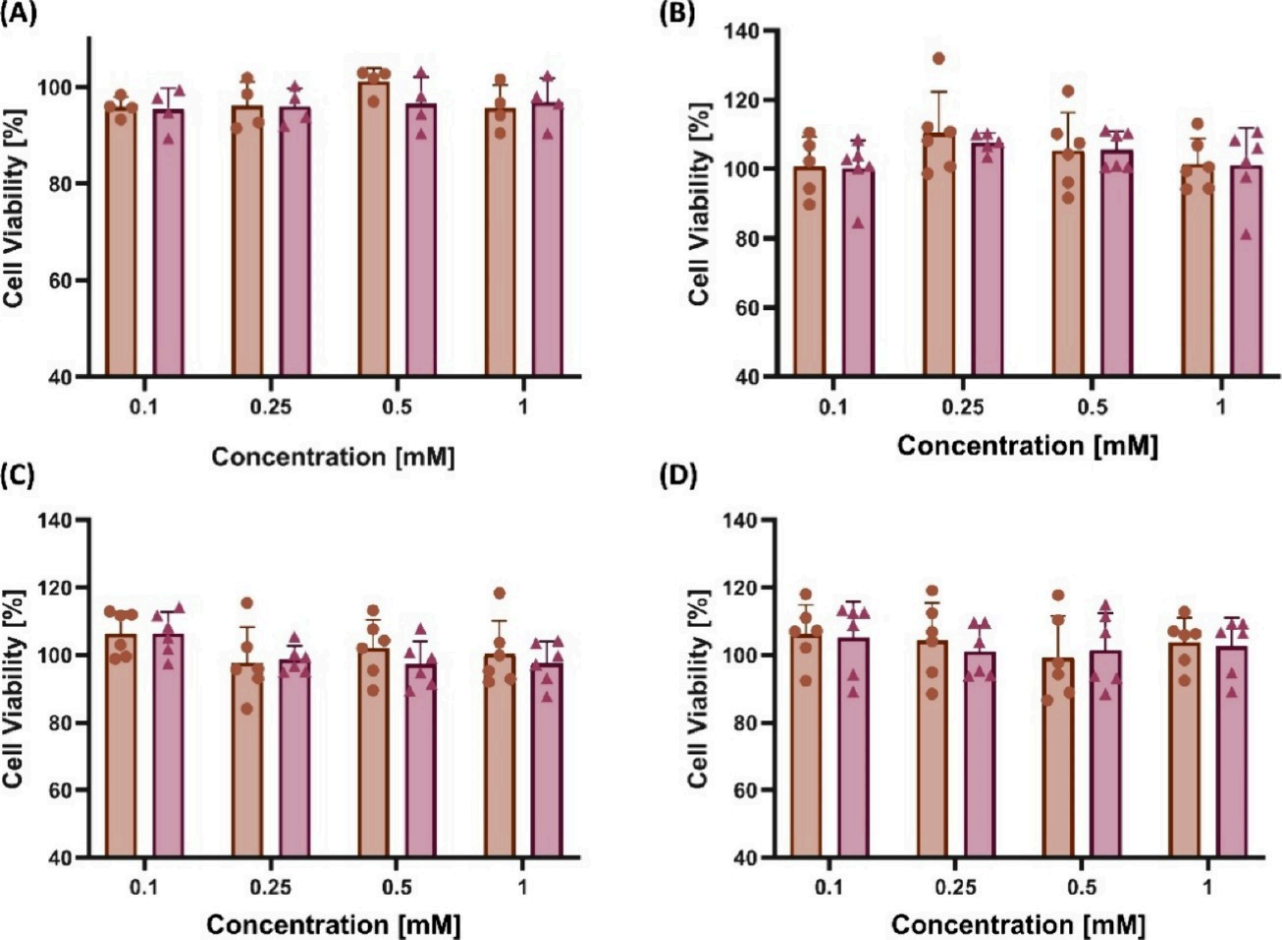
Cytotoxicity testing of MPsomes and liposomes on endothelial monolayer cells, M1-activated macrophages, M2-activated macrophages, and nonactivated macrophages cell line. No MPsomes and liposome groups resulted in significant toxicity up to 1 mM for 60 min post-treatment assessed by the MTT toxicity assay on (A) murine endothelial cells, (B) nonactivated J774 macrophages, (C) LPS-activated M1-like macrophages, and (D) IL4-activated M2-like macrophages.

To further evaluate the biosafety of the NPs formulations, MPsomes and liposomes were tested for endotoxin contamination using a Quant-iT Endotoxin Detection Assay Kit. The analysis revealed a low and nonsignificant difference in endotoxin content between liposomes (0.67 ± 0.04 EU mL^–1^) and MPsomes (0.61 ± 0.04 EU mL^–1^) (Figure S2). These levels are below the endotoxin acceptance limit for intravenous administration in mice, as defined by the United States Pharmacopeia (USP, chapter 85 ^32^) and FDA guidelines,^33^ which establishes the maximum allowable endotoxin concentration using the formula *K*/*M*, where *K* is the threshold pyrogenic dose (5 EU kg^–1^ for intravenous, nonintrathecal administration) and *M* is the maximum dose volume administered per kilogram of body weight. For the present dosing regimen (0.15 mL injected into a 20 g mouse, corresponding to 7.5 mL kg^–1^), the calculated limit is approximately 0.67 EU mL^–1^. Both liposomes and MPsomes are, therefore, within the acceptable range for intravenous administration. Moreover, these values are consistent with those typically reported for nanoparticle formulations used in preclinical research, where measured endotoxin levels generally range between 0.1 and 1 EU mL^–1^, depending on material type and purification strategy.^34^

### 5 MPsomes Demonstrated a Higher Association Level with Inflamed Endothelial Cells *In Vitro*

The TNBC microenvironment is characterized by chronic inflammation, which promotes tumorigenesis.^35,36^ Consequently, we selected endothelial cells for *in vitro* testing of MPsome association and uptake as these are the first inflamed cells likely to be encountered following systemic administration. The highest nontoxic NPs concentration (1 mM) was selected to evaluate NPs’ association with inflamed endothelial cells. Briefly, murine vein endothelial cells were inflamed with LPS and incubated with 1 mM Rhodamine-labeled NPs for 15, 30, 45, and 60 min. Using the Cytation5 device and Gen5 software, we analyzed the association levels by measuring the red fluorescence intensity within a 10 μm radius from the identified cell nuclei (Figure S3). Interestingly, after 15 min of incubation, the association level of MPsomes was 1.4-fold higher than that of liposomes. Furthermore, after 45 and 60 min of incubation, MPsomes demonstrated association levels that were 2.3-fold and 3.1-fold higher, respectively, compared to liposomes (Figure 4A,B). Additionally, the association level of liposomes remained constant over time, while the association level of MPsomes increased by 1.9-fold from 15 to 30 and 45 min and by 2.5-fold from 15 to 60 min of incubation.

**Figure 4:**
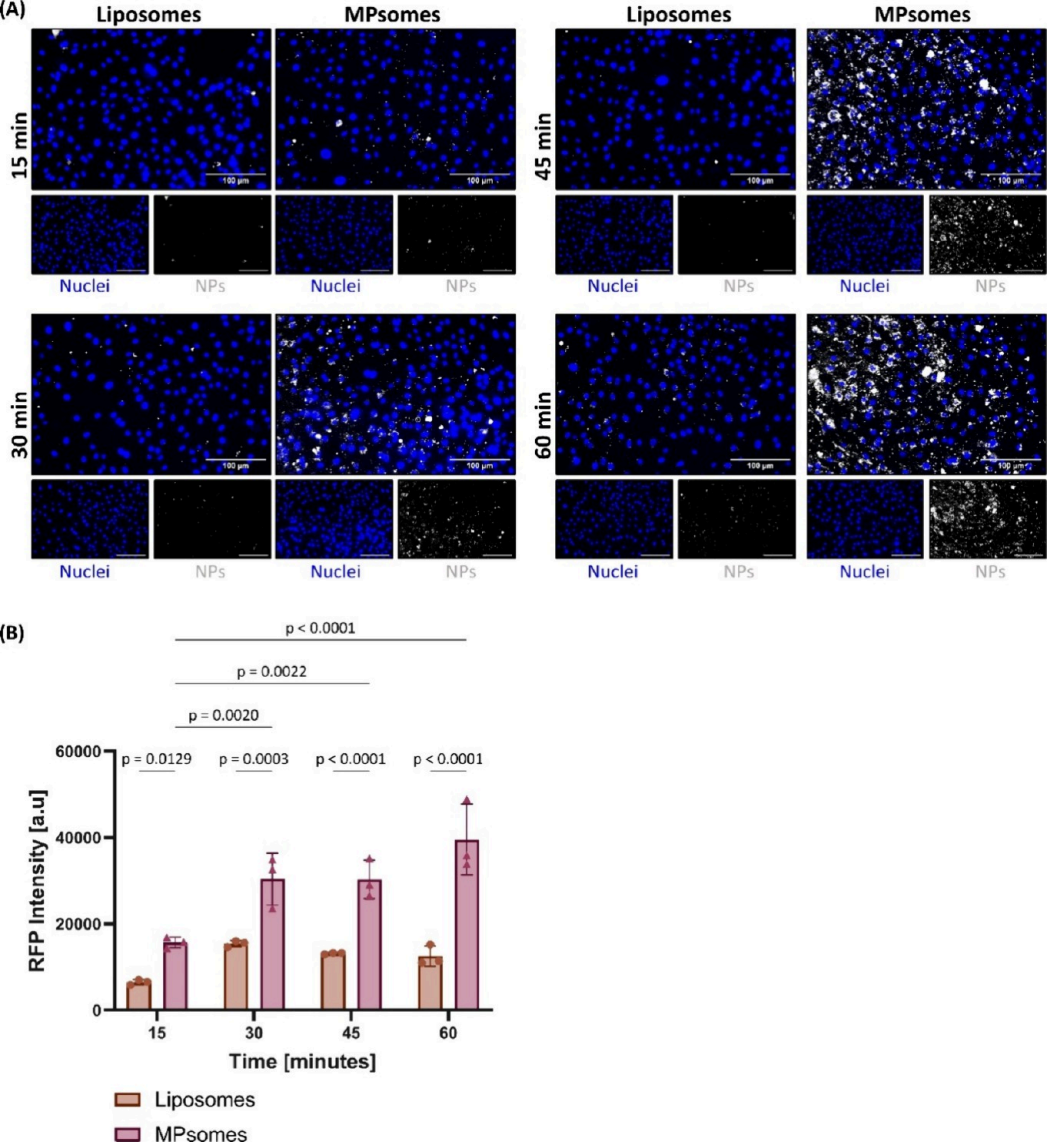
Association evaluation of MPsomes and liposomes on the endothelial monolayer in static conditions for up to 60 min. (A) Cytation5 fluorescent microscope was used to assess both NPs groups’ association with inflamed endothelial cells up to 60 min at predetermined 15 min intervals. (B) Representative images of both NPs groups' association up to 60 min. Red = NPs, blue = nuclei. Scale bar = 100 μm, black and white images represent the red channel. Results are shown as mean ± SD. Two-way ANOVA followed by Tukey’s multiple comparison test was used to determine statistical probabilities. *P* value ≤ 0.05 among means was considered statistically significant.

These results highlight the superior and time-dependent association of MPsomes with inflamed endothelial cells compared with liposomes, emphasizing their potential for enhanced targeting in the TNBC microenvironment.

### 6 MPsomes Compete with Macrophages on the Association with Inflamed Endothelial Sites under Flow Conditions *In Vitro*

Given the higher association ability of MPsomes with inflamed endothelial cells, we further evaluated the capacity of macrophages to adhere to inflamed endothelial cells in the presence of NPs under conditions that mimic blood flow in tumor vessels (Figure 5A). Using a μ-slide I luer 0.4 unit with a syringe pump system, we simulated blood flow in tumor blood vessels. The flow rate was determined according to the μ-slide application note and calculated using the following equation:

$$\Phi [\frac{\mathrm{mL}}{\min}]=\frac{\tau [\frac{\mathrm{dyne}}{{\mathrm{cm}}^{2}}]}{\eta [\frac{\mathrm{dyne}\cdot s}{{\mathrm{cm}}^{2}}]\cdot \mathrm{slide}\;\mathrm{factor}}$$

**Figure 5:**
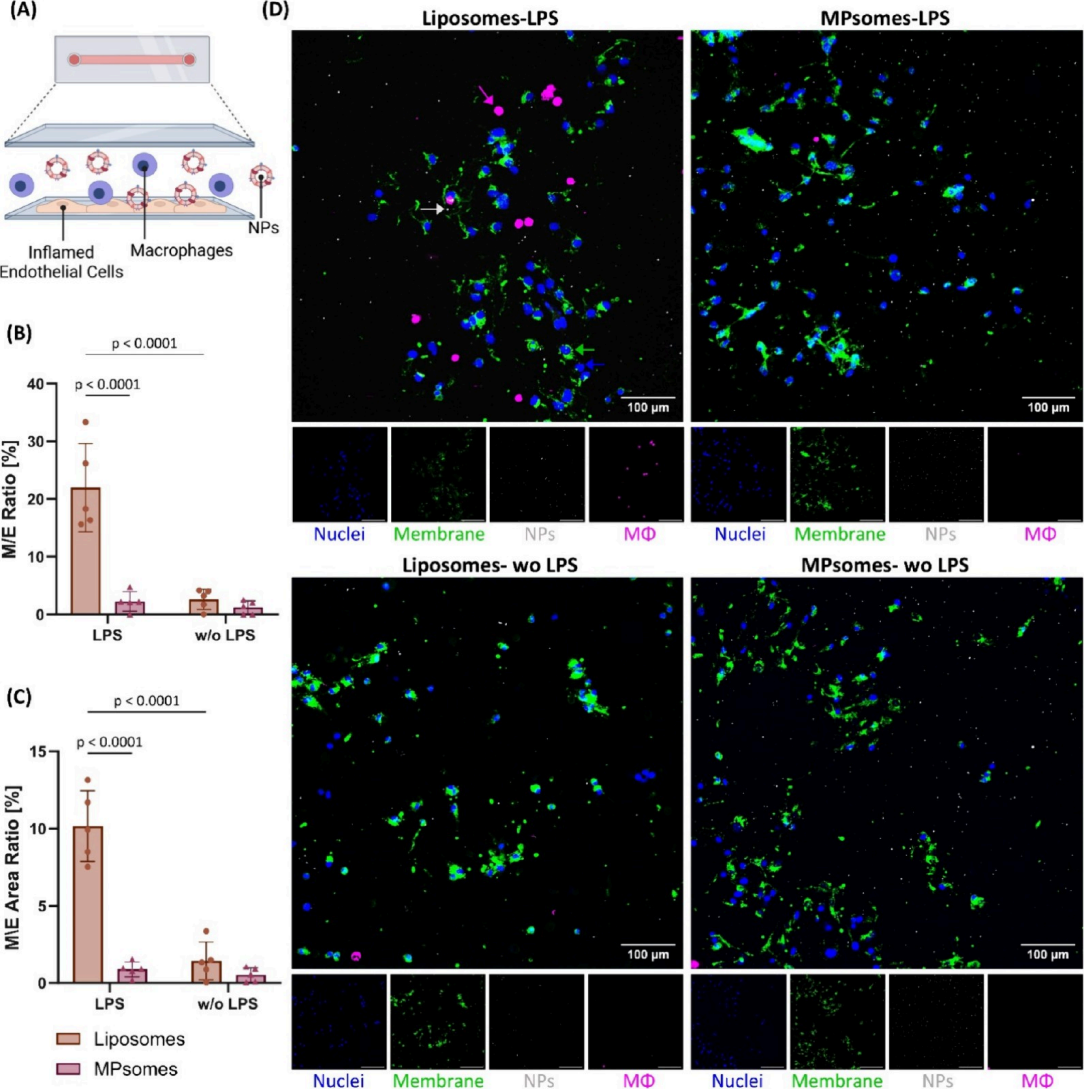
MPsomes outcompeted macrophages in associating with inflamed endothelial sites compared to liposomes in a dynamic assay. (A) Schematic representation of the flow chamber assay used for assessing MPsome–macrophage competition. (B) MPsomes outcompeted macrophages in associating with inflamed endothelial sites compared to liposomes under tumor vasculature-mimicking conditions. (C) The ratio (%) of the area occupied by macrophages (purple channel) to the area occupied by endothelial cells (green channel). (D) Representative fluorescent images captured by a spinning disk confocal device, showing the μ-slide unit under flow conditions with MPsome and liposome-treated slides. Blue = endothelial cells’ nuclei, green = endothelial cells’ membrane, red = NPs, purple = macrophages. Scale bar = 100 μm. To enhance visualization, representative arrows in the relevant colors that indicate the presence of macrophages, NPs, and endothelial cells were added; (C) the ratio of macrophages attached to inflamed endothelial cells to the number of endothelial cells after 1 h of streaming [%].

Here, η represents the dynamic viscosity; τ is the shear stress; and Φ is the flow rate. Elevated interstitial fluid and blood or lymph flow within the primary tumor generate a constant fluid shear stress, ranging from 0.1 to 1 dyn/cm^2^, which can influence cell behavior.^37^ By selecting an average shear stress value of 0.55 dyn/cm^2^ and using the slide factor of 131.6 from the μ-slide I luer 0.4 application note, along with the dynamic viscosity of cell culture medium at 37 °C (0.00785 dyn·s/cm^2^),^38^ we calculated the flow rate to be 0.53 mL/min. Next, a macrophage concentration of 0.3 × 10^5^ cells/mL, based on physiological data from the Mouse Phenome Database (MPD) for 8 week-old Balb/c female mice,^39^ was used along with an NPs concentration of 1 mM.

In this assay, murine vein endothelial cells were seeded in the μ-slide, inflamed with LPS, and imaged under a spinning disk confocal microscope every 3 min for 1 h at 37 °C while streaming culture medium containing 1 mM rhodamine-labeled NPs and 0.3 × 10^5^ cells/mL DiD-labeled macrophages (Figure S4A, Video S1). Since J774 macrophages are adherent cells, we used a 3 mL syringe that was reloaded to ensure that the cells remained viable and did not settle during the streaming (Figure S4B,C).

After 1 h of streaming medium with macrophages and NPs, the slides were washed with PBS at the same flow rate. Then, the number of macrophages adhering to the endothelial membrane was quantified and expressed as the ratio of macrophages to endothelial cells. Interestingly, the ratio of macrophages adhering to inflamed endothelial cells in the presence of MPsomes was 9.7-fold lower than that in the presence of liposomes. Furthermore, no significant increase in the ratio of adhering macrophages to either inflamed or noninflamed endothelial cells was observed in the presence of MPsomes. In contrast, in the presence of liposomes, the ratio of macrophages adhering to inflamed endothelial cells was significantly 8.4-fold higher than that of macrophages adhering to noninflamed endothelial cells (Figure 5B,D). In addition, no significant difference in the ratio of macrophages adhering to healthy, noninflamed endothelial cells in the presence of liposomes or MPsomes was observed.

Area-based analysis confirmed these findings, showing an 11.6-fold higher adhesion ratio with liposomes than MPsomes and a 7.1-fold increase compared to noninflamed cells (Figure 5B–D, Figure S5A–C). Our findings underscore the unique capability of MPsomes to inhibit macrophage adhesion specifically to inflamed endothelial cells, highlighting their potential to mitigate excessive macrophage recruitment under inflammatory conditions.

### 7 MPsomes Influence on Macrophage Migration toward Inflamed Endothelial Cells *In Vitro*

To assess the impact of MPsomes on macrophage recruitment, we used a Transwell migration assay (Figure 6A). Murine vein endothelial cells were inflamed with 100 ng/mL LPS for 24 h, and then macrophages were added to 8 μm inserts and settled for 10 min. NPs (1 mM) were introduced into the intermediate buffer and incubated for 1 h. After removing nonmigrating cells, inserts were fixed, stained with Hoechst, and imaged. Macrophage migration was quantified within a 5.5 mm region using image analysis tools (Figures S6 and S7). Macrophage migration was 2.5-fold higher toward LPS-inflamed endothelial cells compared to noninflamed untreated cells. When NPs were added to the intermediate buffer, MPsomes were 2.4-fold more effective in reducing macrophage migration than liposomes toward inflamed endothelial cells. Specifically, in the presence of MPsomes, the level of macrophage migration toward inflamed endothelial cells was similar to that toward noninflamed endothelial cells. In contrast, in the presence of liposomes, the number of migrating macrophages toward inflamed endothelial cells was 2.6-fold higher than toward noninflamed endothelial cells (Figure 6B,D). We confirmed these findings by calculating the area percentage of macrophages that had migrated across the inset. The results were consistent: the area percentage of migrating macrophages was 3.5-fold higher toward inflamed endothelial cells than noninflamed cells. In the presence of liposomes, the migration to inflamed cells was 3.6-fold higher than that with MPsomes (Figure 6C).

**Figure 6:**
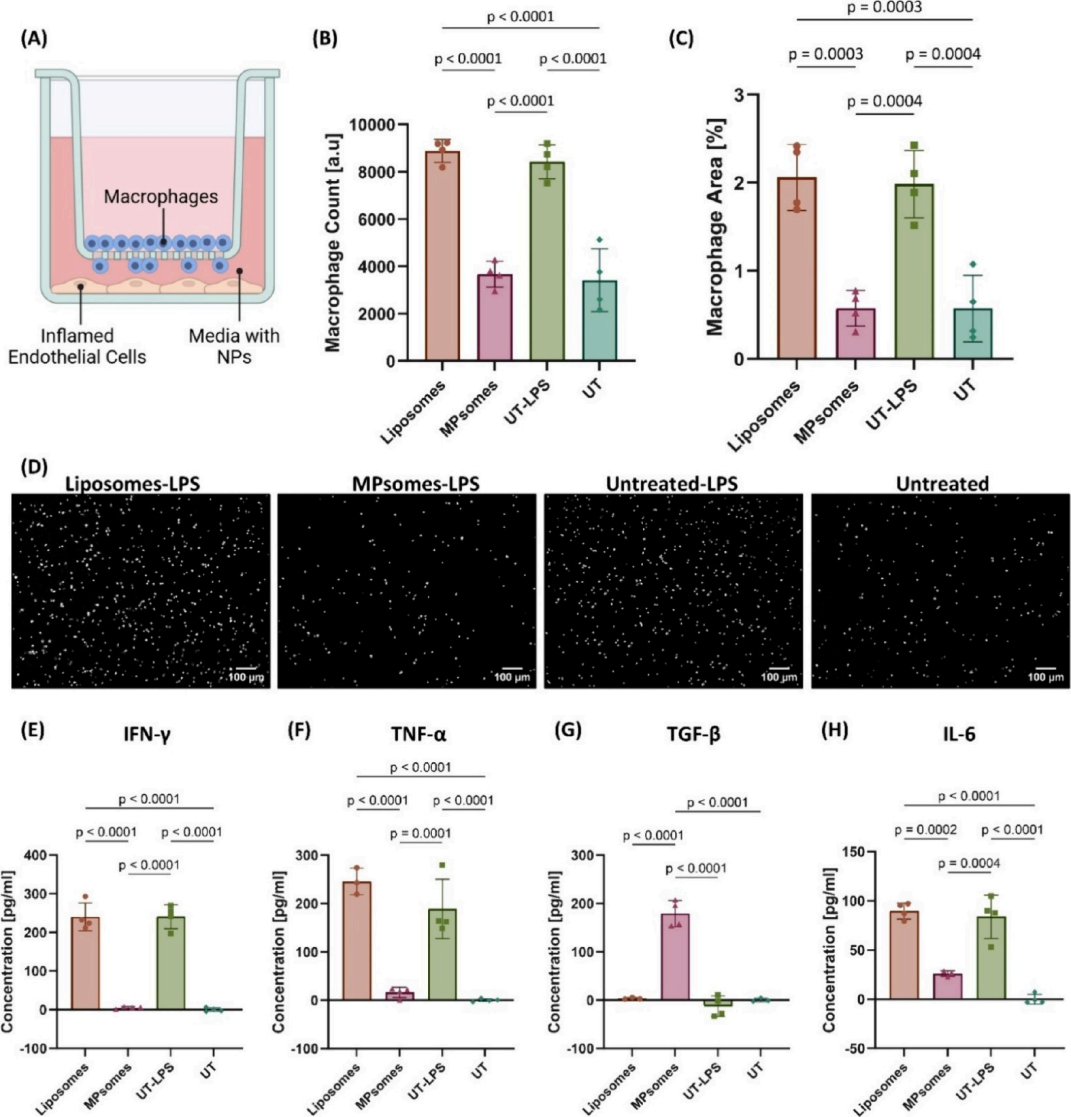
MPsomes reduced macrophage migration more effectively than liposomes in a Transwell static assay. (A) Schematic representation of the Transwell macrophage migration assay model used for inhibition evaluation. (B) The number of migrating macrophages in the Transwell inserts for each treatment group [a.u.]. (C) Percentage of the area occupied by migrating macrophages within the whole insert area (5.5 mm × 5.5 mm) for each treatment group. Each point represents an independent insert, quantified using image analysis tools. (D) Representative images of stained macrophages at the bottom of the Transwell inserts for each treatment group were captured using the Inverted Leica DMI8 microscope. White = macrophage nuclei. Scale bar = 100 μm; the concentrations [pg/mL] of (E) IFN-γ, (F) TNF-α, (G) TGF-β, and (H) IL-6 in the intermediate buffer from the Transwell assay were quantified for each treatment group using ELISA. Statistical analysis was performed using one-way ANOVA followed by Tukey’s multiple comparison test. Data are presented as mean ± SD, with a *p*-value ≤ 0.05 considered statistically significant.

Considering the significant change in the number of migrating macrophages in the presence of MPsomes in the intermediate buffer, we further analyzed the presence of inflammation-related cytokines in this buffer using an enzyme-linked immunosorbent assay (ELISA). Specifically, we quantified the protein levels of interleukin-6 (IL-6), interferon-γ (IFN-γ), tumor necrosis factor-α (TNF-α), and transforming growth factor-β (TGF-β). IFN-γ was undetectable in the untreated and LPS-MPsomes-treated groups, whereas comparable average concentrations of 240.16 and 240.38 pg/mL were observed in the LPS-liposome-treated and LPS-untreated groups, respectively (Figure 6E). Similarly, TNF-α was absent in the untreated and LPS-MPsome-treated groups, while the LPS-untreated group exhibited an average concentration of 158.36 pg/mL and the LPS-liposome-treated group showed a 1.5-fold increase, reaching 245.35 pg/mL (Figure 6F). TGF-β was not detected in the untreated, LPS-untreated, or LPS-liposome-treated groups; however, it was present at an average concentration of 104.83 pg/mL in the LPS-MPsomes-treated group (Figure 6G). Lastly, IL-6 was undetectable in the untreated and LPS-MPsomes-treated groups, while the LPS-untreated group exhibited an average concentration of 83.75 pg/mL and the LPS-liposomes-treated group showed a similar concentration of 89.64 pg/mL (Figure 6H).

These results highlight the superior ability of MPsomes to suppress macrophage migration toward inflamed endothelial cells and modulate the inflammatory microenvironment, as evidenced by their effect on cytokine profiles.

### 8 MPsomes Exhibit Superior Tumor Accumulation and Retention Compared to Liposomes *In Vivo*

TNBC tumors were induced in 8 week-old Balb/c female mice and confirmed for tumor formation by luciferin injection on the 10th day before NPs administration. To evaluate the biodistribution of MPsomes compared to liposomes *in vivo*, both NPs were labeled with Cy5, injected intravenously, and imaged using IVIS at 6, 24, and 48 h postinjection. Fluorescent signal intensities were quantified in tumors and major organs, including the liver, lungs, spleen, heart, kidneys, and blood. MPsomes demonstrated significantly higher accumulation in tumors at all examined time points, showing a 20-fold increase in tumor fluorescence relative to liposomes at 6 h, 15-fold at 24 h, and 9-fold at 48 h postinjection (Figure 7A,B). In filtering organs (liver, kidneys, heart, lungs, and blood), no significant differences in fluorescent intensity were observed between the treatment groups at any time point. However, in the spleen, MPsomes-treated mice exhibited elevated fluorescence levels, with a 1.7-fold increase compared to liposomes at both 6 and 24 h postinjection, while no significant differences were detected at 48 h (Figure 7C–E, Figure S8). Thus, MPsomes can effectively target TNBC tumors while maintaining a biodistribution profile comparable to conventional liposomes.

**Figure 7:**
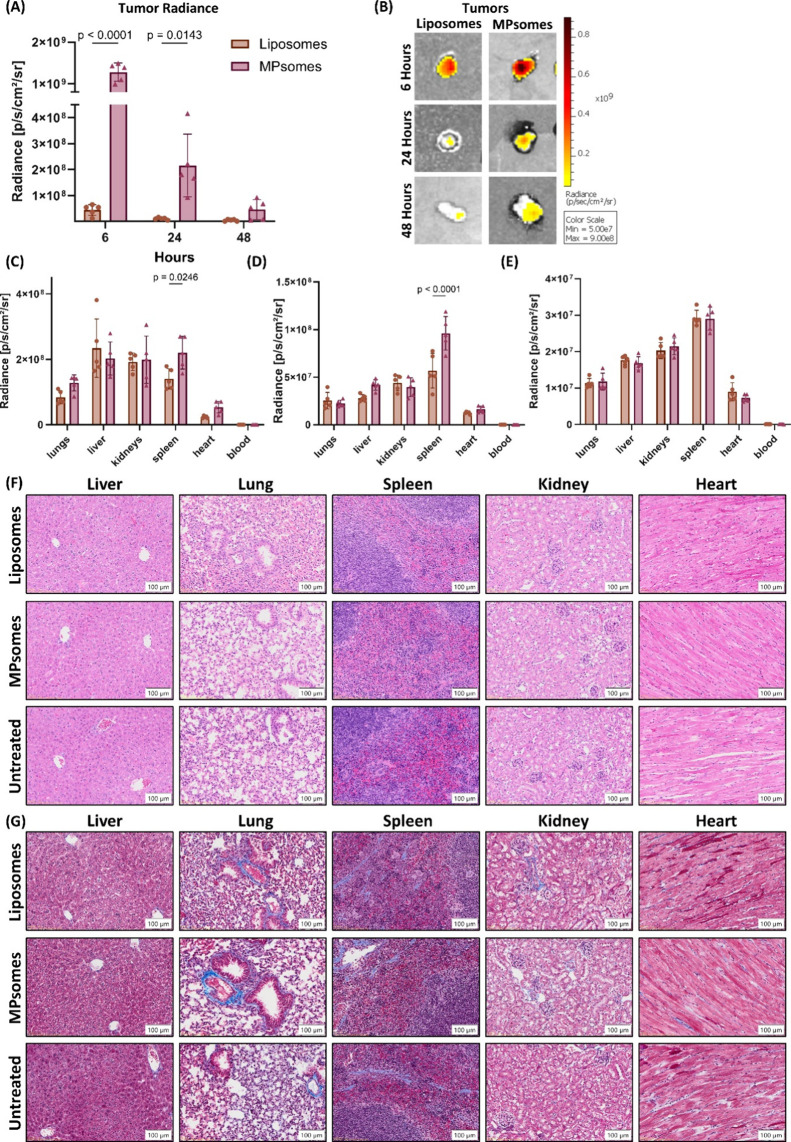
Enhanced tumor accumulation and safety profile of MPsomes in a TNBC murine model. (A) Quantification of Cy5-labeled nanoparticle fluorescence intensity over time showing significantly higher accumulation of MPsomes in the tumor compared to liposomes at 6, 24, and 48 h postinjection, as measured by IVIS. (B) Representative IVIS fluorescence images of the tumors confirming enhanced MPsome retention. (C–E) Relative fluorescence intensity in major organs (liver, spleen, lung, kidney, heart, and blood) at 6, 24, and 48 h postinjection, indicating biodistribution patterns of both formulations. (F) Hematoxylin and eosin (H&E) staining and (G) Masson’s trichrome staining of major organs after 48 h showing no histopathological abnormalities or fibrosis, confirming the safety of MPsomes treatment.

To further evaluate the safety of MPsomes treatment, the liver, spleen, kidneys, lungs, and heart were harvested, sectioned, and stained using hematoxylin and eosin (H&E) (Figure 7F) and Masson’s trichrome (MS) for pathological examination (Figures 7G, S12, and S13). Microscopic analysis revealed no pathological abnormalities in the examined tissues, with normal organ morphology.^40^ Thus, MPsome or liposome administration does not induce detectable structural damage in vital filtering organs.

### 9 MPsomes Reduce Tumor Growth in a Murine TNBC *In Vivo* Model

Given the observed decrease in adherent macrophages to inflamed endothelial cells and the reduction in migrating macrophages toward inflamed endothelial cells in the presence of MPsomes, we proceeded to evaluate MPsomes’ therapeutic efficacy compared to liposomes using a TNBC orthotopic murine model (Figure 8A). Mice were treated with 150 μL of 10 mM MPsomes or liposomes intravenously and administered three times a week for 2 weeks while monitoring their weight, overall behavior, and tumor diameter size using caliper measurements. A control group received 150 μL of saline on the same schedule, and an additional group was treated with anti-PD1 antibody (100 μg per mouse, three times a week for 2 weeks), representing the current gold-standard and FDA-approved immunotherapy for TNBC.^41^ Notably, throughout the experiment, mouse body weight remained stable across all treatment groups, indicating that the treatments did not cause any fluctuations (Figure S9).

**Figure 8:**
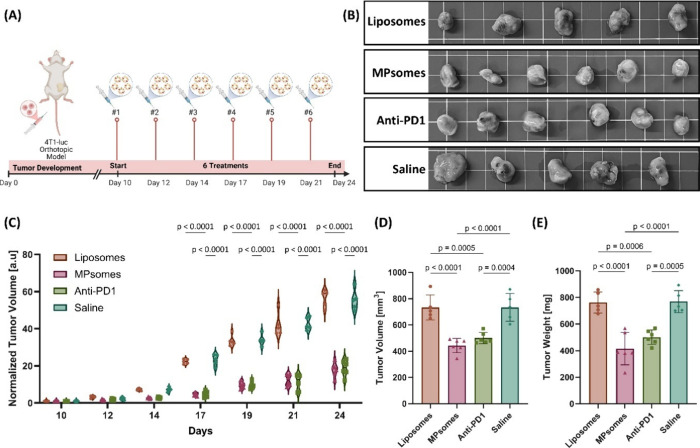
MPsome treatment significantly decreases triple-negative breast cancer tumor growth compared to the liposome-treated group *in vivo*. (A) Schematic illustration of the 4T1-luc orthotopic tumor model, including treatment timeline. (B) Excised tumors from all treatment groups were collected on day 24 and displayed together for visual comparison. (C) Normalized tumor volume calculated based on diameter measurements for all treated groups over 14 days [a.u.]. Tumor volumes for each mouse were normalized to their initial tumor volume at the start of treatment, enabling comparison of growth dynamics across animals with different baseline tumor sizes. (D) Tumor volume [mm^3^] and (E) tumor weight [mg] of the resected tumors on the last day of the experiment, after euthanizing the mice; results are presented as mean ± SD. Two-way ANOVA followed by Sidak’s multiple comparison test was used to determine statistical probabilities. *P* value ≤ 0.05 among means was considered statistically significant.

By normalizing the tumor volume, calculated as a sphere using the measured diameter, of each mouse to its initial tumor volume measured on the 10th day, we observed slower growth in the MPsomes-treated and anti-PD1-treated groups than in the liposome-treated and saline-treated control group. Fascinatingly, the average normalized tumor volume of the MPsome-treated group was 4.8-fold lower on the 17th day than the average normalized tumor volume of the liposome-treated and saline-treated groups, with no significant difference in tumor volume between the MPsome-treated and anti-PD1-treated groups. From this point until the end of the experiment (day 24), this difference increased, and by the final day, the normalized tumor volume in the MPsome-treated and anti-PD1-treated groups was 3- and 2.9-fold lower than in the liposomes-treated and saline-treated groups, respectively (Figure 8B,C). At the end of the experiment (day 24), the weight and volume of the excised tumors in the MPsomes-treated and anti-PD1-treated groups were 1.9-fold and 1.7-fold lower, respectively, compared to those of the liposome-treated and saline-treated groups (Figure 8D,E).

These findings underscore the immunotherapeutic efficacy of MPsomes in reducing tumor growth and size in a TNBC murine model, demonstrating their potential as a more effective treatment strategy compared with conventional liposomes.

### 10 MPsome Treatment Reduces the Percentage of Macrophages and Tumor-Associated Macrophages within the Tumor Tissue, Leading to Slower Tumor Growth *In Vivo*

To further analyze the cause of the slower tumor growth observed in the MPsomes and anti-PD1 groups, the relevant immune cell content within the TME and the spleen tissue was assessed. Tumors and spleens from all groups were dissociated and analyzed using a flow cytometry-based assay using the following antibodies: anti-CD45, anti-CD11b, anti-F4/80, anti-CD3, anti-CD8a, anti-CD4, anti-FoxP3, anti-CD19, anti-Ly6G, and anti-Ly6C. Macrophages were characterized as Zombie–, CD45+, CD11b+, F4/80+; TAMs were defined as Zombie–, CD45+, CD11b+, F4/80+, Ly6C–, and Ly6G–;^42^ monocytic myeloid-derived suppressor cells (M-MSDCs) as Zombie–, CD45+, CD11b+, and Ly6Chigh; polymorphonuclear myeloid-derived suppressor cells (PMN-MDSCs) as Zombie–, CD45+, CD11b+, and Ly6Ghigh; cytotoxic T cells as Zombie–, CD45+, CD3+, and CD8a+; helper T cells were identified as Zombie–, CD45+, and CD4+; regulatory T cells were identified as Zombie–, CD45+, CD4+, and FoxP3+; and B cells were identified as Zombie–, CD45+, and CD19+ (Figure S10). Immune cell proportions were calculated relative to both the total cell population and the total immune cell population (Zombie–CD45+), with the immune cell-based data presented in Figure S10.

Interestingly, we observed a significant reduction in TAMs population within the MPsomes-treated tumors, which displayed lower percentages compared with all other treatment groups. Specifically, TAMs levels were 4-fold higher in liposome-treated tumors, 3.9-fold higher in saline-treated tumors, and 4.3-fold higher in anti-PD1–treated tumors relative to MPsomes (Figure 9A and S11). A similar trend was observed for the broader macrophage population (CD11b+, F4/80+). In the tumor tissue, macrophage levels in the liposome, saline, and anti-PD1 groups were 7.4-, 6.4-, and 6.7-fold higher, respectively, compared to those in MPsomes (Figure 9B). In the spleen, this trend persisted, with macrophages elevated 2.7-, 2.5-, and 2.6-fold in the liposome, saline, and anti-PD1 groups, respectively (Figure 9I). Consistent with these findings, both monocytic (M-MDSCs) and polymorphonuclear MDSCs (PMN-MDSCs) were significantly reduced in MPsome-treated tumors and spleens (Figure 9C–D,J–K). In tumors, M-MDSCs were 2.2-, 2.0-, and 2.2-fold higher in the liposome, saline, and anti-PD1 groups, respectively, while in spleens, these populations were elevated 2.7-, 3.0-, and 2.7-fold. PMN-MDSCs followed the same pattern, with tumors showing 2.2-, 2.2-, and 1.8-fold increases and spleens 1.5-, 1.5-, and 1.4-fold increases over MPsomes.

**Figure 9:**
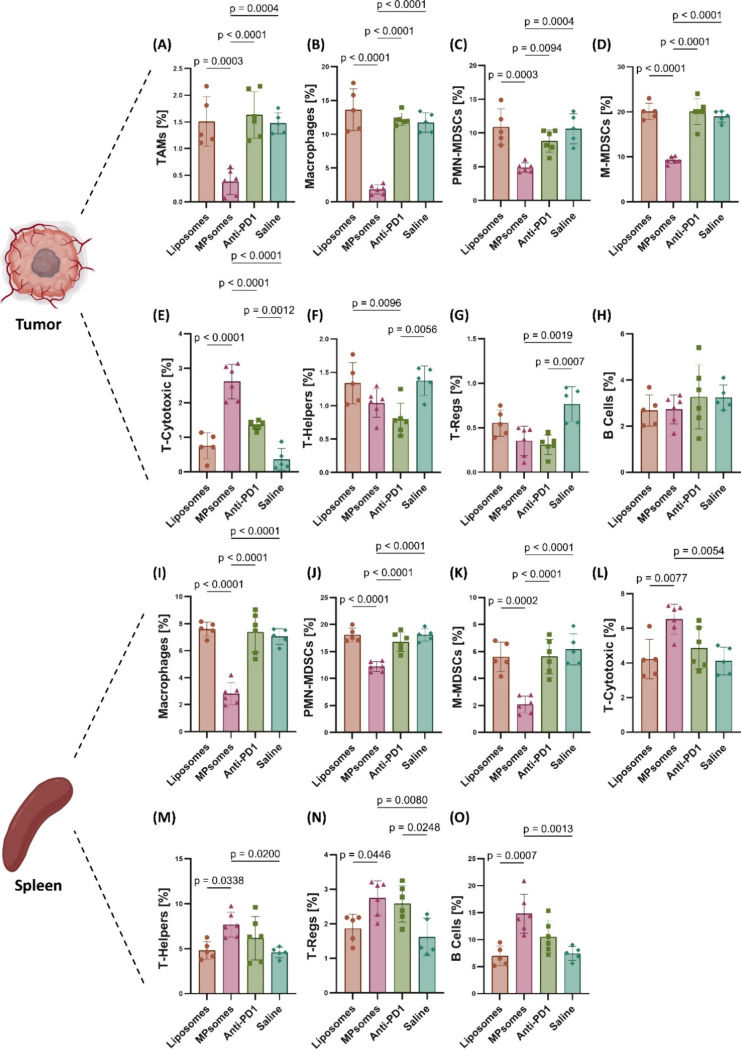
MPsome treatment modulates the immune population within the tumor and spleen tissues and led to a decreased TAMs population in the TNBC tumors compared to liposomes treatment. The percentage of (A) macrophages, (B) TAMs, (C) PMN-MDSCs, (D) M-MDSCs, (E) T-cytotoxic, (F) T-helper, (G) T-regulatory, and (H) B cells among the total tumor cell population, in the groups treated with MPsomes, liposomes, anti-PD1, or saline. (I) Macrophages, (J) PMN-MDSCs, (K) M-MDSCs, (L) T-cytotoxic cells, (M) T-helper, (N) T-regulatory, and (O) B cells among the total spleen cell population. Data are presented as mean ± SD; *p* values indicate statistical significance between groups.

T cell populations demonstrated an opposite trend, as cytotoxic T cells (CD8a+) were significantly enriched in MPsomes-treated tumors, reaching 3.5-, 3.7-, and 1.9-fold higher levels than in the liposome, saline, and anti-PD1-treated groups, respectively. Interestingly, anti-PD1 also enhanced cytotoxic T cells compared to saline with a 3.7-fold increase. In spleens, cytotoxic T cells remained higher in MPsomes compared to all groups (1.6-, 1.7-, and 1.4-fold higher than liposomes, saline, and anti-PD1, respectively) (Figure 9E,L). Helper T cells (CD4+) showed a different pattern: in tumors, both liposome- and saline-treated groups had 1.7-fold higher levels than those of anti-PD1, while MPsomes did not significantly differ from anti-PD1. In the spleens, however, MPsomes yielded 1.6- and 1.7-fold higher helper T cell levels than liposomes and saline, respectively (Figure 9F,M). Regulatory T cells (Tregs) displayed a mixed behavior. In tumors, saline treatment led to significantly higher Treg levels (2.4-fold higher than those of MPsomes and anti-PD1), with no difference between liposome and saline groups. In spleens, MPsomes resulted in increased Tregs, with 1.5-, 1.7-, and 1.6-fold higher levels compared to liposome, saline, and anti-PD1, respectively (Figure 9G,N). Finally, B cells showed no significant differences among the groups in the tumor tissue. However, in the spleens, MPsomes treatment led to an enrichment, with B cells 2.1- and 2.0-fold higher than in the liposome and saline groups and 1.4-fold higher than in the anti-PD1 group (Figure 9H,O).

To further support the flow cytometry results, we conducted immunohistochemistry (IHC) analysis of specific TAMs markers in the tumors. CD163 and CD68 are commonly used TAMs markers in IHC experiments,^43,44^ where elevated levels indicate an increase in TAMs population within the tumor tissue. The IHC images support the flow cytometry results, displaying a lower expression of TAMs markers (CD68, CD163) in the MPsomes-treated tumors than in the liposomes-, anti-PD1-, or saline-treated tumors. Specifically, the DAB-to-hematoxylin ratio in MPsome-treated tumor sections was substantially lower than in all other treatment groups. For CD163 staining, the ratio was 3.7-fold lower compared to liposomes, 3.4-fold lower compared to saline, and 3.8-fold lower compared to anti-PD1-treated sections. For CD68 staining, the ratio was 3.0-fold lower than liposomes, 3.3-fold lower than saline, and 3.3-fold lower than anti-PD1-treated sections. (Figure 10A,B).

**Figure 10:**
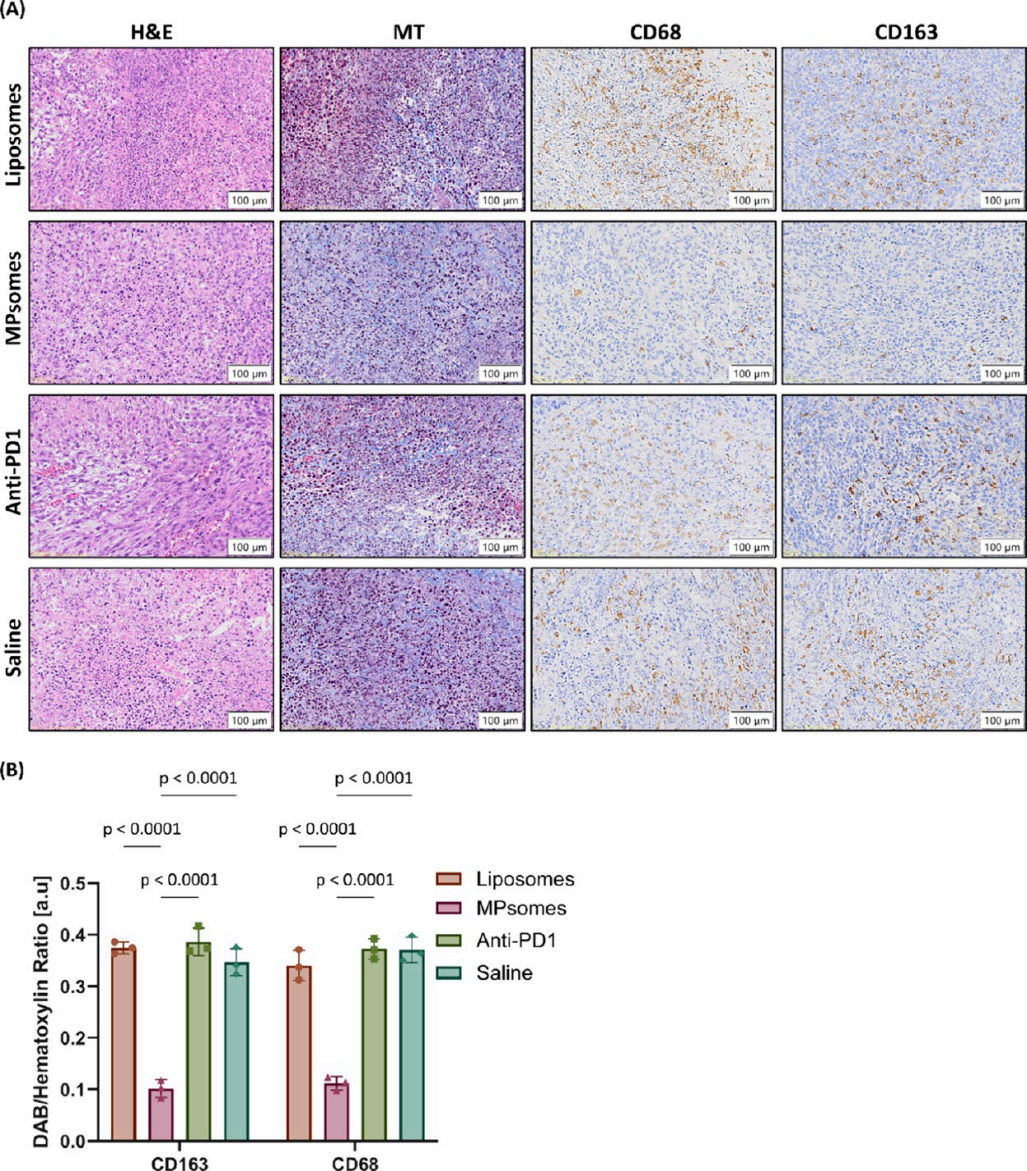
Histological and immunohistochemical analysis of tumor sections following treatment with Liposomes, MPsomes, anti-PD1 or saline. (A) Representative images of tumor sections stained with hematoxylin and eosin (H&E), Masson’s trichrome, and immunohistochemistry for CD68 and CD163 using DAB staining, for each treatment group. Scale bar = 100 μm. (B) Quantification of DAB/hematoxylin ratio performed using a custom Python-based image analysis script for color deconvolution and area quantification of DAB and hematoxylin signals. Results are presented as mean ± SD. Statistical analysis was performed using two-way ANOVA followed by Sidak’s multiple comparisons test. *P* ≤ 0.05 was considered statistically significant.

While biomimetic NPs are traditionally regarded as drug delivery vehicles rather than immunotherapeutic agents, our findings reveal that drug-free, macrophage-mimicking MPsomes can themselves serve as active biological messengers. By leveraging membrane-derived signaling and immune-modulatory interactions, these functional, “empty” MPsomes act as potent antitumor agents in TNBC- demonstrating that therapeutic efficacy can arise not only from delivered drugs but also from the biological communication encoded in the nanoparticle’s surface architecture.

This study demonstrated that the MPsome synthesis process is highly reproducible (Figure 1A–D), consistently producing NPs with uniform physicochemical and biomimetic properties. MPsomes have a more negative zeta potential, indicating the successful integration of membrane proteins, which contributed to their enhanced stability.

Upon serum incubation, MPsomes and liposomes showed changes in their physiochemical properties (Figure 1E–G), likely due to the formation of a protein corona layer. Our findings, consistent with previous studies,^14^ revealed that integrating macrophage membrane proteins into the MPsome bilayer affected the protein corona formation, resulting in significant size growth only after 24 h of incubation. This contrasts with liposomes, which showed a significant size increase after 6 h and a notable change in membrane thickness after 1 h of incubation. This difference may be attributed to the cloaking effect of the integrated proteins on the MPsomes’ surface, reducing nonspecific interactions as was demonstrated by Corbo and others.^14,15^ These structural alterations are expected when NPs encounter serum, as protein adsorption alters their surface composition and hydrodynamic behavior.^14^ However, in the case of MPsomes, the delayed and less pronounced changes likely reflect improved colloidal stability and a more controlled protein corona formation. This behavior suggests that MPsomes maintain structural integrity and surface functionality longer under physiological conditions, which are key factors for achieving sustained circulation and targeted accumulation at inflamed tumor vasculature.

MPsomes contain macrophage-related membrane proteins, as demonstrated by protein profiling through proteomic analysis, qualitative SDS-PAGE, and WB analysis. The high Pearson correlation coefficients obtained from the protein intensities of the replicates indicate a significantly strong correlation, confirming the consistency and reproducibility of the MPsome assembly process. This underscores the potential of MPsomes as a robust biomimetic platform for nanocarriers. Identifying unique macrophage-related membrane proteins within the MPsome fraction can contribute to the macrophage-like functionality of MPsomes. This distinct and consistent pattern was visualized using SDS-PAGE of the NP fraction and WB analysis of CD11b as a representative membrane protein. When comparing total cell lysate proteins, extracted membrane proteins, and MPsome-derived proteins, the WB analysis revealed enrichment of CD11b as a representative marker within the membrane protein fraction compared to the total cell proteins.

Given these findings, we assessed the functional consequences of MPsomes’ interaction with the TNBC microenvironment *in vitro*. When evaluating the ability of MPsomes to modulate the TNBC TME *in vitro*, we found that both MPsomes and liposomes did not induce cytotoxicity at any of the examined nanoparticle concentrations, as determined by an MTT assay performed on endothelial cells, nonactivated macrophages, and macrophages polarized to M1 and M2 phenotypes using LPS and IL-4, respectively. This was expected because the lipids we used (e.g., DPPC, DOPC, and cholesterol) are biocompatible^45^ and the membrane proteins are extracted from murine macrophages. To ensure that the observed cellular responses were not influenced by endotoxin contamination, endotoxin levels in both formulations were quantified using the LAL assay. MPsomes (0.61 ± 0.04 EU mL^–1^) and liposomes (0.67 ± 0.04 EU mL^–1^) displayed comparable and low endotoxin content, close to but not exceeding the calculated regulatory threshold for intravenous administration in mice (0.67 EU mL^–1^, according to USP <85> and FDA guidelines^32–34^ ). These values fall within the range commonly reported for nanoparticle formulations (0.1–1 EU mL^–1^) and indicate that differences in biological performance arise from the nanocarrier composition rather than pyrogenic contamination.

Next, we compared both NPs’ association rates to inflamed endothelia under static conditions. MPsomes exhibit a higher association with tumor-related inflamed endothelial cells than liposomes, indicating enhanced targeting capabilities in the TNBC microenvironment.

To better mimic physiological conditions, we assessed the ability of MPsomes to target inflamed endothelial cells under dynamic conditions. MPsomes significantly decreased the number of macrophages adhering to inflamed endothelial cells under dynamic conditions that simulate tumor vasculature, consistent with their targeting ability observed under static incubation conditions. Thus, the macrophage-related membrane proteins incorporated onto the MPsome surface remain functional under physiological flow conditions, enabling MPsomes to mimic adherence of macrophages to inflamed cells during immune responses. This adherence allows MPsomes to compete with macrophages at inflamed sites, potentially influencing the macrophage recruitment. By inhibiting macrophage adhesion to inflamed endothelial cells, MPsomes effectively disrupt one of the earliest steps in the recruitment of macrophages to the tumor site. Endothelial activation within the tumor vasculature promotes the expression of adhesion molecules such as ICAM-1, VCAM-1, and E-selectin, which facilitate monocyte binding, rolling, and transmigration into tumor tissue.^18,46^ By competing on these adhesion sites, MPsomes reduce the influx of circulating monocytes and macrophages that would otherwise differentiate into TAMs. Consequently, the decreased accumulation of pro-tumorigenic macrophages limits the establishment of an immunosuppressive and pro-angiogenic microenvironment.^18^

The ability of MPsomes to interfere with macrophage recruitment was further validated by using migration assays. Migration assay analysis revealed a reduction in the number of macrophages migrating toward inflamed endothelial cells in the presence of MPsomes in the intermediate buffer compared to liposomes. MPsomes have functional macrophage-related membrane proteins on their surface, allowing them to adhere to specific sites on inflamed endothelial cells, such as macrophages, during an immune response.

These findings are further supported by cytokine analysis, which suggests that MPsomes actively modulate the inflammatory response. Based on the results of the migration assay, MPsomes can also engage in the macrophages and inflamed endothelial cells’ crosstalk,^47^ leading to reduced recruitment of macrophages to these inflamed sites. These results are consistent with the inflammatory cytokine profile revealed by the ELISA analysis. When endothelial cells are treated with LPS, they secrete pro-inflammatory cytokines, such as IL-6,^48^ IFN-γ,^49^ and TNF-α,^50^ which regulate innate immune response and monocyte recruitment. Elevated levels of IL-6, IFN-γ, and TNF-α can indicate an active inflammatory response, promoting macrophage infiltration and further amplifying immune cell recruitment. Our findings demonstrate that the presence of MPsomes in the intermediate buffer resulted in a reduced inflammatory cytokine profile compared to the LPS-liposomes-treated group, supporting the hypothesis that MPsomes can modulate immune signaling, actively suppress the pro-inflammatory environment typically induced by LPS stimulation, and reduce macrophage migration. Interestingly, MPsome treatment led to an increased presence of TGF-β, suggesting a potential anti-inflammatory shift. We observed the presence of TGF-β exclusively in the LPS-MPsomes-treated group, suggesting a potential shift toward an anti-inflammatory or immunosuppressive response. TGF-β is known to regulate immune homeostasis and plays a key role in resolving inflammation by inhibiting macrophage activation and promoting tissue repair.^51^ This shift in the cytokine balance may contribute to the reduced macrophage migration observed in the Transwell assay. Together, these findings suggest that MPsomes not only physically interact with inflamed endothelial cells to reduce macrophage adhesion and migration but also actively modulate the cytokine milieu, potentially attenuating the inflammatory cascade.

While *in vitro* macrophage studies were performed using the J774A.1 murine macrophage-like cell line, we acknowledge that immortalized cells do not fully recapitulate the heterogeneity of primary macrophages. J774A.1 cells were employed for their robustness and widespread use in mechanistic assays of macrophage adhesion, migration, and inflammatory signaling,^52^ enabling controlled comparison between MPsomes and liposomes.

The biodistribution studies demonstrate that MPsomes preferentially accumulate in TNBC tumors compared with conventional liposomes, highlighting their enhanced tumor-targeting capability. MPsomes exhibited markedly higher tumor fluorescence at all examined time points, with a 20-fold increase at 6 h, 15-fold at 24 h, and 9-fold at 48 h postinjection. This enhanced tumor accumulation is likely due to a combination of the enhanced permeability and retention (EPR) effect in tumor vasculature and the biomimetic properties of MPsomes, which allow them to adhere to inflamed endothelium and evade rapid clearance.^13,27^ Accumulation in the liver, kidneys, heart, lungs, and blood was comparable between MPsomes and liposomes, indicating that the increased tumor localization is not accompanied by off-target deposition during the chosen time points. Elevated fluorescence was observed in the spleen at 6 and 24 h in MPsome-treated mice, which may reflect their macrophage-mimicking behavior, facilitating recognition and transient uptake by splenic phagocytic cells, including macrophages and dendritic cells.^53^ Moreover, histopathological analysis of these organs using H&E and Masson’s trichrome staining revealed no detectable abnormalities,^40^ confirming that both MPsomes and liposomes are well tolerated and do not induce structural damage in vital filtering organs. Together, these findings underscore the potential of MPsomes as a safe and efficient nanoplatform for selective delivery to TNBC tumors, achieving high tumor accumulation and maintaining organ integrity.

To determine the *in vivo* therapeutic relevance of MPsomes, we further assessed their impact in a murine TNBC model. MPsomes and anti-PD1 antibody, an FDA-approved immunotherapy, exhibited superior antitumor activity compared to liposomes and saline treatment. Mice treated with either MPsomes or anti-PD1 antibody showed a significant slowdown in tumor growth and a notable reduction in the final tumor volume and weight without affecting the body weight. The antitumor effect of anti-PD1 is attributed to its ability to block the PD-1/PD-L1 immune checkpoint pathway, thereby restoring the function of exhausted T cells and boosting cytotoxic T cell responses.^54–57^ MPsomes, in contrast, can adhere to tumor-related inflamed vasculatures and block the adhesion of infiltrating macrophages, as demonstrated in our *in vitro* models, thereby reducing the number of macrophages infiltrating the tumor and potentially affecting its growth. Supporting that, flow cytometry analysis revealed a significant reduction in the percentages of macrophages and TAMs only within the tumor tissue of MPsomes-treated mice compared to the anti-PD1-, liposomes-, and saline-treated groups. This decrease was confirmed by immunohistochemistry (IHC) analysis, which revealed lower expression levels of TAMs markers (CD68 and CD163) in the MPsomes-treated tumors. Thus, MPsomes retain their macrophage-like activity *in vivo*, reaching the tumor and inhibiting the recruitment of macrophages, especially TAMs, by adhering to inflamed endothelial cells. This blocks the ability of endogenous macrophages to attach to these inflammation sites. As a result, there are fewer infiltrating macrophages in the tumor, which makes it more challenging for the tumor to grow and evade the immune system.

Interestingly, beyond TAMs, MPsome treatment also reshaped other immune cell populations in both tumor and spleen tissues. Macrophages and myeloid-derived suppressor cells (M-MDSCs and PMN-MDSCs) were consistently lower in MPsome-treated mice compared to liposome-, saline-, and anti-PD1–treated groups, likely due to MPsomes’ ability to mimic leukocytes and block recruitment of these immunosuppressive myeloid cells to the TME. In contrast, MPsomes promoted cytotoxic T cell (CD8+) enrichment in both tumors and spleens, exceeding levels observed in all other treatment groups, including anti-PD1. This may be a consequence of reduced myeloid-mediated immunosuppression and enhanced antigen presentation, creating a more favorable environment for T cell activation.^58^ As previously reported, anti-PD1, an FDA-approved immunotherapy, is established for boosting CD8+ T cell activation and restoring function in exhausted T cells.^54,57^ Thus, MPsome treatment can replicate these effects, highlighting their potential as complementary or alternative immunotherapy. Helper T cells (CD4+) displayed a similar pattern, with MPsomes and anti-PD1 treatment increasing their presence in the spleens and tumors compared with saline and liposome groups. The increase following MPsome treatment may be due to reduced immunosuppressive myeloid populations, such as TAMs and MDSCs, which indirectly promote CD4+ T cell expansion in secondary lymphoid organs like the spleen.^58^ In the case of anti-PD1, the effect is likely driven by checkpoint blockade-mediated reactivation of exhausted T cells, which enhances both helper and cytotoxic T cell proliferation and function in systemic and tumor compartments.^54,57^ Regulatory T cells (Tregs), in contrast, were reduced in tumors following MPsomes and anti-PD1 treatment compared to saline. This reduction may result from MPsomes decreasing immunosuppressive myeloid populations and limiting signals that recruit or expand Tregs within the tumor microenvironment,^58^ while anti-PD1 may indirectly suppress Treg activity by restoring effector T cell function and altering cytokine balances.^57,59^ Interestingly, MPsomes also expanded B cell populations in the spleen relative to all other groups, which may be due to enhanced antigen availability and presentation following reduced myeloid-mediated suppression,^60^ leading to humoral immune activation. No significant differences were observed in tumors, suggesting that this effect is primarily systemic rather than local.^61,62^

Taken together, these findings demonstrate that MPsomes not only reduce the infiltration of immunosuppressive macrophages and TAMs but also broadly remodel the tumor immune microenvironment, enhancing cytotoxic T cell activity and reducing suppressive myeloid and Treg populations. This immune reprogramming replicates some of the known benefits of anti-PD1 therapy while adding unique effects on macrophage and B cell regulation. Such results suggest that MPsomes hold promise as a novel immunotherapeutic platform, either as a standalone approach or in combination with FDA-approved immune checkpoint inhibitors like anti-PD1.

While MPsomes are derived from macrophage membranes, it is important to distinguish them from naturally secreted macrophage-derived exosomes. Exosomes are nanosized vesicles formed through the endosomal pathway and contain a complex, cell-specific cargo of proteins, lipids, and nucleic acids that can influence the recipient cell behavior through horizontal biomolecule transfer. However, exosome composition is inherently heterogeneous and difficult to control, varying significantly among cell batches, culture conditions, and isolation methods. Moreover, exosome purification is technically challenging and often yields preparations with inconsistent purity and bioactivity.^24,63^ In contrast, MPsomes are synthetic liposome-based nanoparticles engineered through the controlled integration of macrophage membrane proteins into a defined lipid bilayer. This design allows for precise control over composition, reproducibility, and scalability while preserving macrophage-like surface functionalities such as adhesion and immune-modulatory interactions. Importantly, MPsomes do not carry endogenous nucleic acids or uncontrolled intracellular cargo, thereby minimizing off-target or unpredictable biological effects.^13^ Taken together, these distinctions underscore the advantages of MPsomes as a well-defined, drug-free, biomimetic immunotherapeutic platform capable of reshaping the tumor microenvironment and limiting TAMs recruitment. A direct comparative analysis between MPsomes and natural exosomes would be scientifically valuable but lies beyond the scope of the current study, which focuses on establishing MPsomes as a biomimetic, drug-free strategy for TNBC treatment compared to the nontreated group, liposomes treated group, and anti PD-1 treated group.

## Conclusions

This study establishes MPsomes as a robust and versatile drug-free biomimetic immunoengineered platform capable of reprogramming the tumor microenvironment in TNBC. Through the controlled integration of macrophage membrane proteins into synthetic lipid bilayers, MPsomes exhibit selective affinity for inflamed endothelium, effectively limit macrophage recruitment, and modulate immune dynamics within the tumor. Unlike antibody-based checkpoint inhibitors, MPsomes are drug-free, cell-membrane-derived nanoparticles that are easier and more cost-effective to fabricate, exhibit superior storage stability, and do not require recombinant protein production or cold-chain handling.^56^

While biomimetic nanoparticles are traditionally regarded as passive drug delivery systems, our findings reveal that macrophage-mimicking MPsomes can themselves function as active biological messengers. By leveraging membrane-derived signaling and immune-modulatory interactions, these functional, “empty” NPs act as potent antitumor agents, demonstrating that therapeutic efficacy can arise not only from delivered drugs but also from the biological communication encoded within the nanoparticle’s surface architecture. Importantly, our biodistribution and safety analyses confirmed preferential tumor accumulation and minimal off-target toxicity, supporting the translational feasibility of the MPsomes. Although the current preclinical dosing regimen involved repeated administration every 2–3 days to maintain robust systemic exposure, future clinical translation would involve optimization based on pharmacokinetics and pharmacodynamics, likely requiring fewer doses over extended intervals.

Moving forward, personalizing MPsomes for patient-specific immune profiles and evaluating their efficacy in diverse preclinical models will be key steps toward clinical application. Furthermore, future research should explore how the polarization state of macrophages used as a membrane source influences MPsome functionality, including comparisons between M0-, M1-, and M2-derived MPsomes.

Together, these findings, as far as we know, position MPsomes as a first-in-class, drug-free immunotherapeutic biomimetic platform, an active nanotechnology capable of reshaping the tumor microenvironment.

## Methods/Experimental Section

### Materials

The following materials and reagents were used in this study: Membrane Protein Extraction Kit (ProteoExtract), acetone, chloroform, methanol, DMSO, Tween 20, phosphate-buffered saline (PBS), RPMI-1640, DMEM, Eukitt­(R) Quick-hardening mounting medium, 25 mm Sterile Syringe Filters, 0.02 μm PVDF, and cholesterol from Sigma-Aldrich-Merck; 1,2-dipalmitoyl-*sn*-glycero-3-phosphocholine (DPPC), 1,2-dioleoyl-*sn*-glycero-3-phosphocholine (DOPC), and 1,2-dipalmitoyl-*sn*-glycero-3-phosphoethanolamine-*N*-(lissamine rhodamine B sulfonyl) (PE-Rhod) from Avanti Polar Lipids, Inc.; dialysis tubes (12–14 kDa and 1000 kDa) from Repligen; Dynamic Light Scattering (DLS), ZetaSizer Nano, and disposable cuvettes for zeta potential measurements from Malvern Instruments; rabbit anti-CD11b (MAB11242–100) for Western blotting, and DuoSet ELISA kits for TNF-α, TGF-β, IFN-γ and IL-6 from R&D Systems; rabbit anti-CD68 (ab125212), rabbit anti-CD163 (ab182422), goat antirabbit IgG-HRP (ab6721), hematoxylin and eosin kit, and DAB substrate kit from Abcam; flow cytometry antibodies: anti-CD45-FITC (BLG-157608), anti-CD11b-Alexa Fluor 647 (BLG-101218), anti-F4/80-APC-Cy7 (BLG-157315), anti-Ly6G-BV711 (BLG-127643), anti-Ly6C-PE (BLG-128008), anti-CD3-Spark Blue 574 (BLG-100276), anti-CD8a-Alexa Fluor 594 (BLG-100758), anti-CD4-BV510 (BLG-100449), anti-FoxP3-Pacific Blue (BLG-126410), anti-CD19-PE-Cy7 (BLG-152418), and Zombie Violet Viability Kit from BioLegend; semi microvolume disposable polystcytokinesyrene cuvettes for size measurements and MycoStrip Mycoplasma Detection Kit from Tamar Ltd.; 5X Sample Buffer from A2S; Trans-Blot Turbo Mini PVDF membrane, TC20 Automated Cell Counter and slides, and Clarity Western ECL Substrate from Bio-Rad Laboratories; Quick Coating Solution from AngioProteomie; NanoAssemblr Benchtop and Microfluidic Cartridge from Precision Nanosystems (Cytiva); Infinite M Plex multimode microplate reader from Tecan; Pierce BCA kit,1-Step TMB ELISA Substrate Solutions, Vybrant DiD Cell-Labeling Solution, Halt protease inhibitor cocktail, Quant-iT Endotoxin Detection Assay Kit (Q32892) and wheat germ agglutinin-Alexa Fluor 488 from ThermoFisher Scientific; μ-slide 0.4 was purchased from ibidi, and Endothelial Cell Medium with supplements from ScienCell; *InVivo*mAb antimouse PD-1 (CD279) (BE0146–5A) from BioXCell.

### Cell Culture

Murine J7741.A macrophages (ATCC) were cultured in high-glucose DMEM supplemented with 10% FBS and 1% penicillin-streptomycin. Murine vein endothelial cell lines (CellBiologics) were cultured in a complete endothelial cell medium containing 5% FBS, 1% endothelial cell growth supplement (ECGS), and 1% penicillin-streptomycin. Murine 4T1-luc TNBC cells (PerkinElmer) were cultured in RPMI-1640 supplemented with 10%FBS, 1% penicillin-streptomycin, and 1% l-glutamine.

### Protein Extraction and Quantification

J774 macrophages were lysed using the ProteoExtract Protein Extraction Kit (Sigma), and protein concentration was measured using the BCA Protein Assay Kit (Pierce, ThermoFisher). Briefly, J774 cells were detached, centrifuged at 300*g* for 10 min at 4 °C, and resuspended in 5 mL of PBS twice. The pellet was then resuspended in 8 mL of extraction buffer I with 80 μL of Halt protease inhibitor cocktail (Thermo Scientific) and incubated at 4 °C for 10 min followed by centrifugation at 16,000*g* for 15 min. The resulting pellet was resuspended in 0.5 mL of extraction buffer II with 5 μL of protease inhibitor cocktail, incubated at 4 °C for 30 min, and centrifuged again at 16,000*g* for 15 min. The supernatant containing membrane proteins was transferred to a new tube, snap-frozen with liquid nitrogen, and stored at −80 °C. Protein concentration was quantified using the Pierce BCA Protein Assay kit. A calibration curve was prepared using albumin diluted in 1 L of PBS to concentrations ranging from 0 to 2000 μg/mL. The membrane protein solution was diluted 1:10 (v/v) in PBS. A volume of 20 μL of each sample was loaded in triplicate into a 96-well microplate and mixed with 200 μL of BCA reagent (reagents A and B mixed 50:1 v/v). The plate was incubated in the dark for 30 min, and absorbance was measured at 562 nm using an Infinite M Plex multimode microplate reader (Tecan).

### Biomimetic NPs Assembly

MPsomes and liposomes were assembled using a NanoAssemblr (Cytiva). For the organic lipid phase, DPPC, DOPC, and cholesterol were dissolved in ethanol in a final molar ratio of 4:3:3. For Rhodamine labeling, 50 μL of Rhodamine-PE (16:0, 1 mg/mL) phase per 1 mL of formulation was added to the organic mixture prior to assembly. The aqueous phase included a 1:40 protein-to-lipid ratio (w/w) diluted in PBS for MPsomes and PBS alone for liposomes. The organic phase was sonicated at 45 °C for 5 min, while the aqueous phase containing the membrane proteins was heated to 42 °C for 1 min. Aqueous and organic phases, in volumes of 667 and 333 μL, respectively, were loaded into 1 mL syringes and connected to the microfluidic cartridge. NanoAssemblr parameters were set to a flow ratio of 2:1, flow rate of 2.5 mL/min, final volume of 1 mL, and temperature of 42 °C, with start and end waste volumes of 0.3 and 0.05 mL, respectively, as per the device manual. The NPs formulations were collected in 15 mL tubes and immediately transferred to 12–14 kDa dialysis bags for liposomes and 1000 kDa dialysis bags for MPsomes. All samples were dialyzed overnight at 4 °C under stirring in 1000× volume of PBS, with the external buffer replaced after 1 and 3 h.

### Biomimetic NPs Physiochemical Characterization and Storage Stability Tests

After synthesis, the NPs were filtered through a 0.22 μm filter and their physicochemical characteristics were determined. Size distribution, polydispersity index (PDI), and zeta potential of the NPs were measured by using a Dynamic Light Scattering Zetasizer Nano ZS (Malvern Panalytical). For size and PDI measurements, 495 μL of 0.02 μm filtered- PBS and 5 μL of NPs sample were mixed in a DTS0012 cuvette, and two measurements were performed. For zeta potential, 900 μL of Milli-Q water, 90 μL of 0.02 μm-filtered PBS, and 10 μL of NPs sample were mixed and transferred to a DTS1070 zeta cuvette for replicate measurements. The NPs were stored in PBS at 4 °C, and characterization was repeated using the Zetasizer Nano ZS after 1, 4, 7, 14, and 21 days. The membrane thickness of the NPs was determined from the cryo-TEM images using ImageJ software, with each data point representing the average of five measurements per particle.

### Cryo-TEM Imaging

The NPs samples were prepared, vitrified, and imaged at the Center for Electron Microscopy of Soft Matter (Technion). NPs samples were diluted 1:5 to a final volume of 30 μL. Using the Leica Automatic Plunge Freezer EM GP2 (Leica Microsystems), samples were prepared at a controlled temperature of 25 °C and 100% relative humidity to prevent evaporation. A 3 μL drop of the diluted NPs sample was placed on a carbon-coated grid (Ted Pella), and excess fluid was removed by automatic blotting. The specimen was then vitrified by plunging it into liquid ethane at its freezing point. The frozen grids were transferred to an FEI Talos 200C High-Resolution TEM microscope (Thermo Fisher Scientific) using the Gatan 626 cryo-holder (Gatan). Imaging was conducted in low-dose mode using a FEI Falcon III direct-imaging camera and a ″volta phase-plate″ to enhance image contrast by converting phase contrast into amplitude contrast.

### NPs Proteomic Analysis

To quantitatively evaluate the reproducibility of the MPsome fabrication process, we prepared three replicates of MPsomes and isolated proteins using acetone precipitation followed by identification via liquid chromatography mass spectrometry (LC-MS/MS). 200 μL of each formulation was precipitated in 80% cold acetone, and then the pellet was air-dried and dissolved in 8.5 M urea, 400 mM ammonium bicarbonate, and 10 mM dithiothreitol (DTT). The proteins were reduced, modified, and digested to small peptides followed by LC-MS/MS running. The protein intensities identified from each replicate were compared, and the reproducibility was assessed using the Pearson correlation coefficient. A comprehensive protein profiling analysis was conducted on three replicates of MPsomes and one replicate of bare liposomes, which served as a control. For the analysis, nondetectable values were replaced with the smallest detectable value, and this value was subsequently subtracted from all data points.

### SDS-PAGE Analysis

Proteins from the NPs fractions were dissolved using a methanol–chloroform gradient to remove lipids, and the concentrations of all samples were adjusted to uniform levels before loading. 900 μL of NPs samples were divided into 150 μL aliquots. Each aliquot received 400 μL of methanol and was vortexed thoroughly, and then 300 μL of chloroform was added and vortexed again. The aliquots were centrifuged at 14,000*g* for 1 min, forming three layers (an aqueous layer on top, a protein flake in the interphase, and a chloroform layer at the bottom). The top layer was removed, 400 μL of methanol was added, and the mixture was vortexed and centrifuged at 20,000*g* for 5 min. The supernatant was discarded, and the protein pellets from all vials were combined and resuspended to a final volume of 70 μL. Protein concentrations were measured using the Pierce BCA Protein Assay Kit to ensure that each sample contained an equal total protein concentration. All protein samples were loaded to an 8% acryl-amid gel, and the gel was run using the Mini-PROTEAN Tetra Vertical Electrophoresis Cell (Bio-Rad) at 100 mV for 1 h. The resulting gel was stained with Coomassie Blue (SimplyBlue SafeStain, Invitrogen) for 1 h, then washed overnight with 20% NaCl (w/v) solution, and imaged using the Chemidoc device.

### CD11b Western Blot (WB) Analysis

The samples for WB analysis included J774 total cell lysate, J774 extracted membrane protein fraction, and proteins from MPsomes and liposomes. Samples were loaded onto 8% acrylamide gel in a final volume of 50 μL. For MPSOMES and liposomes, proteins were isolated using the methanol–chloroform method described above. For the J774 total cell lysate, J774 cells were detached, and 10^6^ cells were transferred to a 15 mL tube and centrifuged at 400*g* for 3 min. The pellet was resuspended in a lysis buffer (150 mM NaCl, 1% Triton X-100, 50 mM Tris, pH 8), incubated for 10 min at 4 °C, and centrifuged at 20,000*g* for 20 min at 4 °C. The resulting protein-containing supernatant was used for WB sample preparation. Before loading, protein concentrations were measured using the Pierce BCA Protein Assay Kit and adjusted to ensure the same amount of protein in each band.

The gel was run using the Mini-PROTEAN Tetra Vertical Electrophoresis Cell (Bio-Rad) at 100 mV for 1 h and transferred to a Trans-Blot Turbo Mini 0.2 μm PVDF membrane (Bio-Rad) using the Trans-Blot Turbo Transfer System (Bio-Rad). The membrane was blocked with 15 mL of 5% skim milk in PBST (PBS + 0.1% Tween) for 1 h at room temperature with gentle agitation. It was then incubated overnight at 4 °C with 15 μL of rabbit anti-CD11b (0.5 μg/mL). After washing three times with 10 mL of PBST, the membrane was incubated with 15 mL of 1:10,000 horseradish peroxidase (HRP)-conjugated goat antirabbit diluted in PBST for 45 min at room temperature. The membrane was washed three times with 10 mL of PBST, incubated for 1 min with 2 mL of 1:1 clarity Western peroxide reagent and clarity Western luminol/enhancer reagent (Bio-Rad), and imaged using the ChemiDoc MP Imaging System (Bio-Rad). Membrane analysis was performed using ImageLab software.

### Stability at 37 °C in FBS

The stability of the NPs’ physicochemical properties over 24 h was investigated. Specifically, 75 μL of NPs sample was diluted in 300 μL of 50%FBS to achieve a final lipid concentration of 2.25 mM. Samples were incubated at 37 °C for 1, 6, and 24 h. Following incubation, all samples were ultracentrifuged at 100,000*g* for 1 h. The supernatant was removed, and the NPs pellet was resuspended in 75 μL of 0.02 μm-filtered PBS. The resulting NPs samples were measured using a Zetasizer Nano ZS (Malvern Panalytical) and imaged with cryo-TEM as previously described. The membrane thickness of the NPs was measured from the cryo-TEM images by using ImageJ software, with each point representing an average of five measurements per particle.

### Endotoxin Evaluation in NPs Formulations

Endotoxin content in liposome and MPsome formulations was determined using the Quant-iT Endotoxin Detection Assay Kit (Thermo Fisher Scientific, MAN0029394) following the manufacturer’s protocol. Samples were diluted in endotoxin-free water to ensure measurements within the linear detection range (0.01–10 EU mL^–1^, depending on the assay volume). A standard curve was prepared from serial dilutions of the *E. coli* O111:B4 endotoxin reference, and fluorescence was measured at 490/525 nm (excitation/emission) using a microplate reader. Endotoxin concentrations were calculated by interpolation from the standard curve after log-transformation and linear regression of the corrected fluorescence values.

### Viability MTT Assay

The cytotoxicity of all NPs on endothelial cells was assessed using the MTT assay. This assay relies on the metabolic activity of live cells, which convert (3-(4,5-dimethylthiazol-2-yl)-2,5-diphenyl tetrazolium bromide) (MTT) into colored formazan through a reduction process, which is measurable by absorbance. Murine vein endothelial cells and J774 macrophages (10,000 per well) were seeded in a 96-well plate in a complete cell medium and grown until 80% confluent. J774 cells were then activated with either 100 ng/mL LPS or 10 ng/mL IL-4 diluted in complete medium for 24 h. Subsequently, varying concentrations of NPs (0.1, 0.25, 0.5, and 1 mM) were added, and the cells were incubated at 37 °C with 5% CO_2_. After incubation, the medium was replaced with a medium containing 0.5 mg/mL MTT salt, and cells were incubated again at 37 °C with 5% CO_2_ for 2 h. The medium was then replaced with dimethyl sulfoxide (DMSO) and incubated at room temperature under gentle agitation for 30 min. Absorbance at 570 nm was measured using the Infinite M Plex multimode microplate reader (Tecan), with a reference wavelength of 630 nm. Viability percentages were calculated relative to the untreated control cells.

### *In Vitro* Association with Inflamed Endothelial Cells

Murine endothelial cells (10,000 per well) were seeded in a 96-well plate with a complete endothelial cell medium and grown until 80% confluent. The medium was then replaced with fresh medium containing 100 ng/mL lipopolysaccharide (LPS) and incubated at 37 °C with 5% CO_2_ for 24 h. Subsequently, the medium was replaced with fresh medium containing 1 mM rhodamine-labeled NPs, and the cells were incubated for 15, 30, 45, and 60 min at 37 °C with 5% CO_2_. Following the incubation, the medium was replaced with warm PBS containing 1:10,000 Hoechst 33342, and the cells were incubated for 15 min. After washing once with warm PBS, the cells were fixed with 4% paraformaldehyde (PFA) in PBS for 20 min at room temperature. Postfixation, the PFA was removed and replaced with 50 μL of PBS. The cells were imaged using Cytation5 (Biotek), and the association level was analyzed using Gen5 software.

### J774 Viability under Dynamic Flow Conditions

Given that J774 macrophages are adherent cells, their viability and quantity were verified using the TC20 Automated Cell Counter (Bio-Rad) to ensure they did not settle during the flow experiment. Cells were detached and adjusted to a concentration of 0.3 × 10^5^ cells/mL in a 40 mL stock solution, which was continuously mixed and reloaded into 3 mL syringes as needed. The output liquid was sampled at 0, 10, 20, 30, 40, 50, and 60 min of flow. Samples were diluted 1:1 (v/v) with trypan blue and counted using a TC20 Automated Cell Counter (Bio-Rad).

### *In Vitro* Flow Chamber Competition Assay

100,000 murine endothelial cells were seeded in a μ-slide 0.4 (ibidi) with 150 μL of complete endothelial cell medium and incubated at 37 °C with 5% CO_2_ until confluence. The cells were then treated with 100 ng/mL LPS in 150 μL of complete endothelial cell medium and incubated for 24 h at 37 °C with 5% CO_2_. After incubation, the medium was replaced with warm PBS followed by the addition of 5 μg/mL WGA-AF488 solution and a 20 min incubation at 37 °C. Cells were washed with warm PBS, and then Hoechst 33342 (1:10,000 dilution) was added and incubated for 15 min at 37 °C. After a final wash with warm PBS, fresh warm medium was added.

J774 macrophages were detached and counted, adjusting their concentration to 0.3 × 10^5^ cells/mL in a 40 mL stock. Cells were centrifuged, the supernatant was removed, and 1 mL of Vybrant DiD Cell-Labeling Solution (1:200 dilution in warm PBS) was added. After a 15 min incubation at 37 °C, cells were centrifuged at 300*g* for 4 min, the supernatant was removed, and 1 mL of fresh medium was added.

A 40 mL stock solution containing 1 mM rhodamine-labeled NPs, 0.3 × 10^5^ cells/mL DiD-labeled J774 macrophages, and phenol red-free endothelial cell medium was prepared. The stock solution was mixed continuously and loaded into a 3 mL syringe in repeated loads. The syringe pump was set to 0.53 mL/min for forward pumping, and 3 mL syringes were replaced using an infusion pump to avoid bubbles. The μ-slide was imaged during the flow using the Spinning Disk Confocal (Nikon) for 1 h at 37 °C. After the flow period, the stock solution was replaced with warm PBS, and the μ-slide was washed at the same flow rate for 3 min. The μ-slide was then imaged again using the same parameters. The macrophage-endothelial cell association level was quantified using ImageJ software.

### *In Vitro* Transwell-Based Macrophage Migration Assay

150,000 murine endothelial cells per well were seeded in a 24-well plate and grown in a complete endothelial cell medium until 80% confluent. The medium was then replaced with medium containing 100 ng/mL LPS, and the cells were incubated at 37 °C with 5% CO_2_ for 24 h. Subsequently, the medium in all wells was replaced with 540 μL of fresh endothelial cell medium. J774 cells were detached, and 100 μL of a 10^6^ cells/mL solution was added to the upper part of ThinCerts TC Inserts 8 μm (Greiner). The plate was incubated for 10 min at 37 °C with 5% CO_2_ to allow the cells to settle. Then, 60 μL of either NPs or PBS (for the untreated group) was added to the intermediate buffer, resulting in a final NPs concentration of 1 mM. The plate was incubated for 1 h at 37 °C with 5% CO_2_. After incubation, the cells on the upper part of the inset were removed using a cotton-tipped applicator, and the cells on the bottom part of the insets were fixed with 70% ethanol at room temperature for 15 min. The membranes were then separated from the insets, stained for nuclei with Hoechst 33342 diluted 1:10,000 in PBS solution at room temperature for 15 min, and washed twice with PBS. Inserts were glued to microscope slides and imaged using the Leica DMI8 inverted fluorescent microscope (Leica). The number of migrating macrophages was determined by using ImageJ software.

### Enzyme-Linked Immunosorbent Assay (ELISA)

MaxiSorp 96-well plates (Thermo Fisher Scientific) were coated with 100 μL of the provided capture antibody for each cytokine (DuoSet ELISA kits: IFN-γ DY485–05, TGF-β DY1679–05, TNF-α DY410, and IL-6 DY406–05, R&D Systems) and incubated overnight at room temperature. Following incubation, the plates were washed and blocked for 1 h at room temperature. Subsequently, 100 μL of intermediate buffer from the Transwell migration assay of the untreated, LPS-untreated, LPS-MPSOMES-treated, and LPS-liposomes-treated groups were added to the wells and incubated for 2 h at room temperature. The plates were then washed three times and incubated with 100 μL of the provided detection antibody for each cytokine for 2 h at room temperature. After another three washes, 100 μL of a 1:40 diluted streptavidin-HRP solution was added to each well and incubated for 20 min at room temperature. The reaction was then developed using 100 μL of 3,3′,5,5′-tetramethylbenzidine (TMB) substrate (Thermo Fisher Scientific) until a blue color appeared, stopped with 1 N H_3_PO_4_, and immediately read at 450 nm with a reference measurement at 540 nm using a plate reader. Cytokine concentrations were determined using standard-based calibration curves. Since some values were negative, we used the untreated (UT) group as a reference and added its average concentration to all other samples. This adjustment minimized negative values while maintaining the variance between replicates.

### *In Vivo* TNBC Model

All animal experiments were performed following the protocols evaluated and approved by the Technion’s Pre – Clinical Research Authority, (Ethics Approval Number: IL0760525). The TNBC model was established by injecting 3 × 10^5^ 4T1-luc (PerkinElmer) cells, suspended in 50 μL of PBS, subcutaneously into the mammary fat pad of 8 week-old BALB/c female mice (Inotiv). 10 days after the tumor cell injection, tumor size was verified using luminescence imaging and caliper measurements before NPs injection. Mice were injected intraperitoneally with luciferin (10 mg/kg) and imaged 10 min postinjection using IVIS SpectrumCT (PerkinElmer).

For biodistribution and biosafety assessment following NPs administration, 150 μL of Cy5-labeled NPs were injected via retro-orbital intravenous delivery. Fluorescence intensity was evaluated *in vivo* at 6, 24, and 48 h postinjection using the IVIS SpectrumCT imaging system. At each time point, major organs were harvested, sectioned, and stained with hematoxylin and eosin (H&E) and Masson’s trichrome for pathological evaluation.

For efficacy evaluation of NPs treatment, 150 μL of NPs, saline, or 100 μg of anti-PD1 antibody in 150 μL of saline was administered via retro-orbital intravenous injection, with caliper measurements and IVIS imaging performed on days 10, 12, 14, 17, 19, and 21. 24 days after tumor cell injection, the tumors were measured with a caliper and imaged using luminescence imaging. The mice were then sacrificed, and the tumors were harvested, weighed, measured using a caliper, and kept for further macrophage population analysis.

### Flow Cytometry Analysis of Resected Tumors

The resected tumors were dissociated using a Tumor Dissociation Kit (Miltenyi Biotec). Tumors were cut into 2–4 mm pieces and mixed with the kit enzyme mix by adding 2.35 mL of RPMI 1640, 100 μL of Enzyme D, 10 μL of Enzyme R, and 12.5 μL of Enzyme A into a gentleMACS C Tube. The C Tube was then attached to the gentleMACS Octo Dissociator with Heaters device (Miltenyi Biotec) and the program “37C_m_TDK_2” was run. After completion, the cells were transferred to a 70 μm strainer placed on a 50 mL tube, washed with 10 mL of RPMI, and centrifuged for 7 min at 300*g*, 4 °C. The resected spleens were mechanically dissociated plunger end of a sterile syringe against a 70 μm cell strainer (Corning) into 5 mL of RPMI-1640. The resulting cell suspension was centrifuged at 400*g* for 5 min at 4 °C, and the supernatant was discarded.

Then, for spleen and tumors, the supernatant was removed, and the cells were resuspended in 1 mL of eBioscience 1× RBC Lysis Buffer (Invitrogen), incubated for 2 min at RT under gentle agitation, and centrifuged for 3 min at 400*g*, 4 °C. The supernatant was removed and replaced with 1 mL of PBS. Next, 2 μL of Zombie Violet Fixable Viability Kit was added, and the cells were incubated for 20 min at room temperature in the dark and then centrifuged for 3 min at 400*g*, 4 °C. The pellet was resuspended in 500 μL of blocking solution (10% FBS in PBS, v/v), incubated for 30 min at 4 °C under gentle agitation, and centrifuged for 3 min at 400*g*, 4 °C. The pellet was resuspended in 100 μL of antibody mix (anti-CD45-FITC, anti-CD11b-Alexa Fluor 647, anti-F4/80-APC-Cy7, anti-Ly6G-BV711, anti-CD3-Spark Blue 574, anti-CD8a-Alexa Fluor 594, anti-CD4-BV510, anti-CD19-PE-Cy7, and anti-Ly6C-PE) in staining buffer (2% BSA, w/v), incubated for 30 min at 4 °C under gentle agitation, and centrifuged for 3 min at 400*g*, 4 °C. The pellet was then resuspended in 500 μL of staining buffer and centrifuged for 3 min at 400*g*, 4 °C, twice. The cells were fixed in 500 μL of 4% paraformaldehyde (PFA) at RT for 20 min, washed in staining buffer, and permeabilized in 1 mL of permeabilization buffer (0.1% Saponin (w/v)+ 1% BSA (w/v)) at RT for 20 min. Finally, the cells were intracellularly stained with 100 μL of anti-FoxP3-Pacific Blue diluted in permeabilization buffer for 30 min at RT, washed twice with permeabilization buffer, resuspended in 500 μL of staining buffer, and analyzed using the Aurora (Cytek) device. FlowJo version 10.10 software was used for analysis to identify macrophage and TAMs populations.

### Immunohistochemistry of Tumor and Organ Sections

Half of a tumor and relevant filtrating organs from the experimental groups was fixed in 4% paraformaldehyde (PFA) and transferred to the Biomedical Core Facility Center at the Technion for histological processing and Hematoxylin and Eosin (H&E) or Masson’s Trichrome staining. At the facility, samples underwent dehydration and clearing using a tissue processor (Leica TP1020, Germany) followed by paraffin embedding with the Histocore Arcadia H system (Leica). Paraffin blocks were sectioned at 4 μm thickness using a rotary microtome (Leica RM2265, Germany). Sections were floated on a 37–39 °C water bath, mounted onto adhesive glass slides (Leica X-tra), and dried overnight at 37 °C. Before staining, sections were deparaffinized in xylene, rehydrated through a graded ethanol series, and stained with either hematoxylin and eosin (H&E) or Masson’s Trichrome. Slides were then cover-slipped using a Leica Histocore Spectra coverslipper. The stained slides were scanned using the Slide Scanner Slideview VS200 at the Biomedical Core Facility center (Technion). For CD163 and CD68 tumor tissue staining, the slides were deparaffinized in xylene, rehydrated through a graded ethanol series at RT. For antigen retrieval, slides were placed in a microwave-safe jar containing Tris-EDTA (TE) buffer (pH suitable for CD68 and CD163) and microwaved at high power until boiling for 4 min followed by 10 min at low power to maintain gentle boiling. The TE buffer was replaced with boiling DDW, and slides were cooled at room temperature for 1.5 h. The slides were then blocked with 10% FBS in PBS (v/v) for 1 h. After removing excess blocking solution, primary antibodies were applied overnight at RT in a humidified chamber: anti-CD68 (1:100, 0.5 μg/mL; stock 0.5 mg/mL) and anti-CD163 (1:200; stock 0.7 mg/mL). The following day, slides were washed three times with PBS and incubated with HRP-conjugated goat antirabbit secondary antibody (1:1000) for 1 h at RT. After washing, 3,3′-diaminobenzidine (DAB) staining was performed by mixing 30 μL DAB chromogen with 1.5 mL of substrate and applying it for up to 10 min, until optimal color development was achieved. Sections were washed with distilled water, counterstained with hematoxylin for 2 min, and treated with a bluing reagent (buffered lithium carbonate) for 10–15 s. Finally, slides were rinsed with distilled water, mounted with Eukitt quick-hardening medium, and air-dried for 20–30 min before imaging using the Slide Scanner Slideview VS200 at the Biomedical Core Facility center (Technion).

### Statistical Analysis

All data are presented as means ± standard deviation (SD). Statistical significance between groups was determined using GraphPad statistical software, with a *P* value ≤ 0.05 considered statistically significant.

## Supplementary Material





## Notes

Additional experimental details and data supporting the main text, including: quantitative analysis of CD11b Western blot results (Figure S1); endotoxin quantification of nanoparticle formulations (Figure S2); Gen5 Cytation5 image analysis workflow for nanoparticle association (Figure S3); flow stability experiments for J774 macrophages (Figures S4 and S5); Transwell migration assay analysis (Figures S6 and S7); *ex vivo* biodistribution of Cy5-labeled nanoparticles (Figure.S8); normalized body weight monitoring (Figure S9); gating strategy and immune cell population analysis in tumor and spleen (Figures S10 and S11); and histological evaluation of major organs at 6 and 24 h post-treatment (Figures S12 and S13) (PDF)
Video showing macrophage streaming on untreated or LPS-treated endothelial cells under flow conditions (Video S1) (MP4)
