# Supplementary material for: Drug-free Immunotherapeutic Biomimetic Nanoparticles for Treating Triple-Negative Breast Cancer
Source: ACS Nano. 2026 Feb 13;20(8):7001–21. doi: 10.1021/acsnano.5c18774 (PMC12961927; doi:10.1021/acsnano.5c18774)
Supplement: Supplementary file 1 [file nn5c18774_si_001.pdf]

Supplementary Information

Drug-free Immunotherapeutic Biomimetic Nanoparticles for Treating Triple-Negative Breast Cancer

Ofri Vizenblit[1], Rawan Mhajne[1], Assaf Zinger[1,2,3,4,5,6,*]

[1]Bioinspired Nano Engineering and Translational Therapeutics Lab, Department of Chemical Engineering, Technion−Israel Institute of Technology, Haifa 3200003, Israel
[2]Russell-Berrie Nanotechnology Institute, Technion – Israel Institute of Technology, Haifa, 3200003, Israel
[3]Resnick Sustainability Center of Catalysis, Technion−Israel Institute of Technology, Haifa 3200003, Israel
[4]Bruce and Ruth Rappaport Cancer Research Center, Technion−Israel Institute of Technology, Haifa 3200003, Israel
[5]Cardiovascular Sciences Department, Houston Methodist Academic Institute, Houston, TX 77030, United States
[6]Neurosurgery Department, Houston Methodist Academic Institute, Houston, TX 77030, United States

(A)

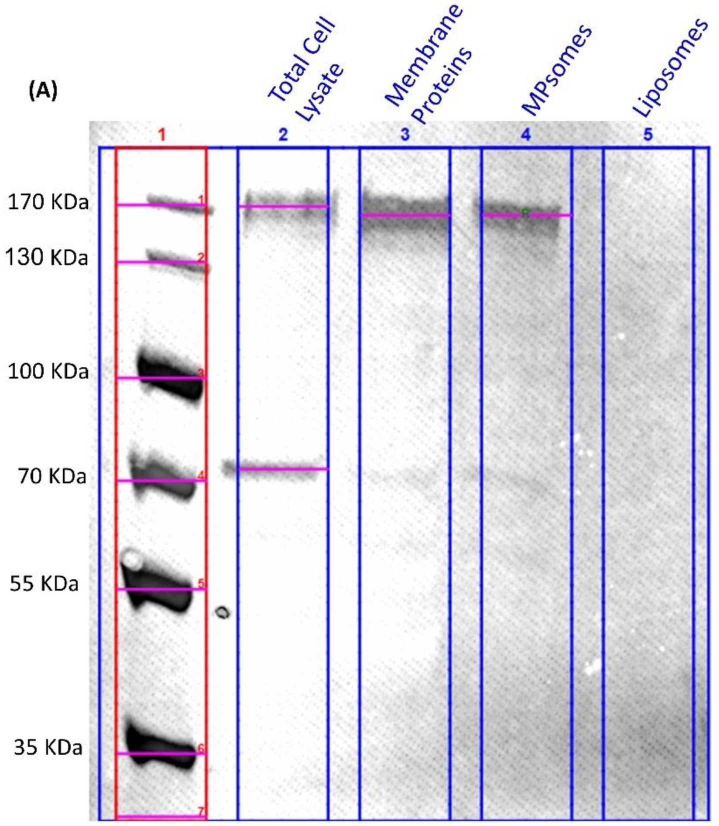

(B)

Lane	Rel. Quant.
2	**1.596095**
2	**0.774303**
3	**1.989333**
4	**1**

Figure S1. Quantitative analysis of CD11b western blot results. (A) Membrane image taken using the ChemiDoc Device (BioRad), with lanes and bands identification using the ImageLab software, the band identified on the MPsomes line was set as the reference band (marked as 'R'); **(B)** Relative bands quantification raw results achieved using the ImageLab software.

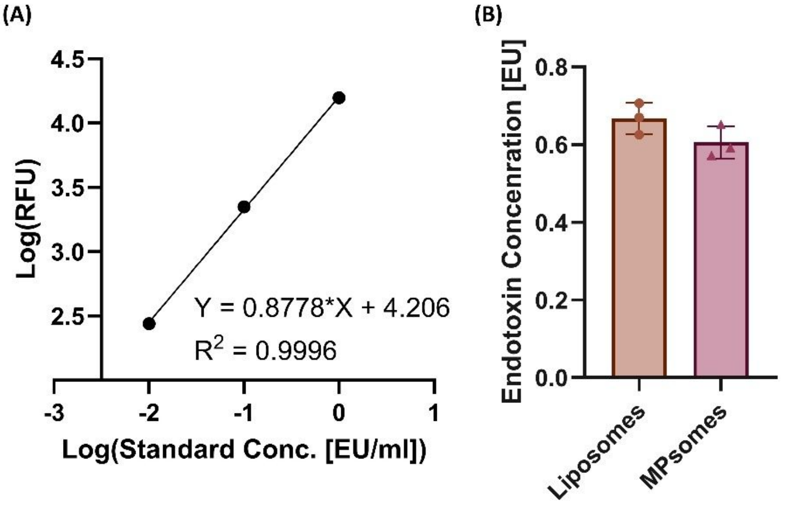

Figure S2. Endotoxin quantification in NPs formulations. (A) Calibration curve of endotoxin standards plotted on a logarithmic scale, used to determine endotoxin concentrations. **(B)** Quantification of endotoxin levels expressed as EU/mL in liposome and MPsomes formulations, showing both samples are within the acceptable range for *in vivo* applications.

(A)

(B)

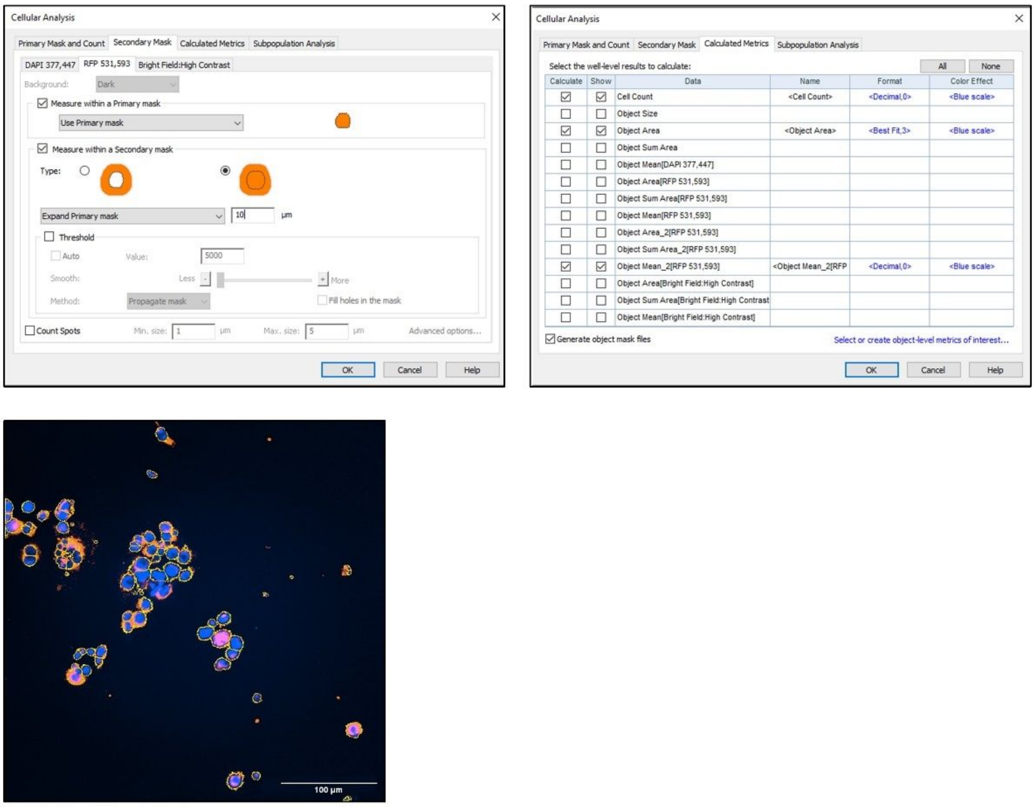

Figure S3. Gen5 Cytation5 analysis algorithm. (A) NPs association levels were quantified using the Gen5 cellular analysis feature. The algorithm initially identified the cell nuclei, followed by the calculation of mean RFP intensity within a secondary mask of 10 µm radius. **(B)** Representative image of the analysis, displaying the secondary mask in yellow. Blue = endothelial cells' nuclei, and red = nanoparticles. Scale bar = 100 µm.

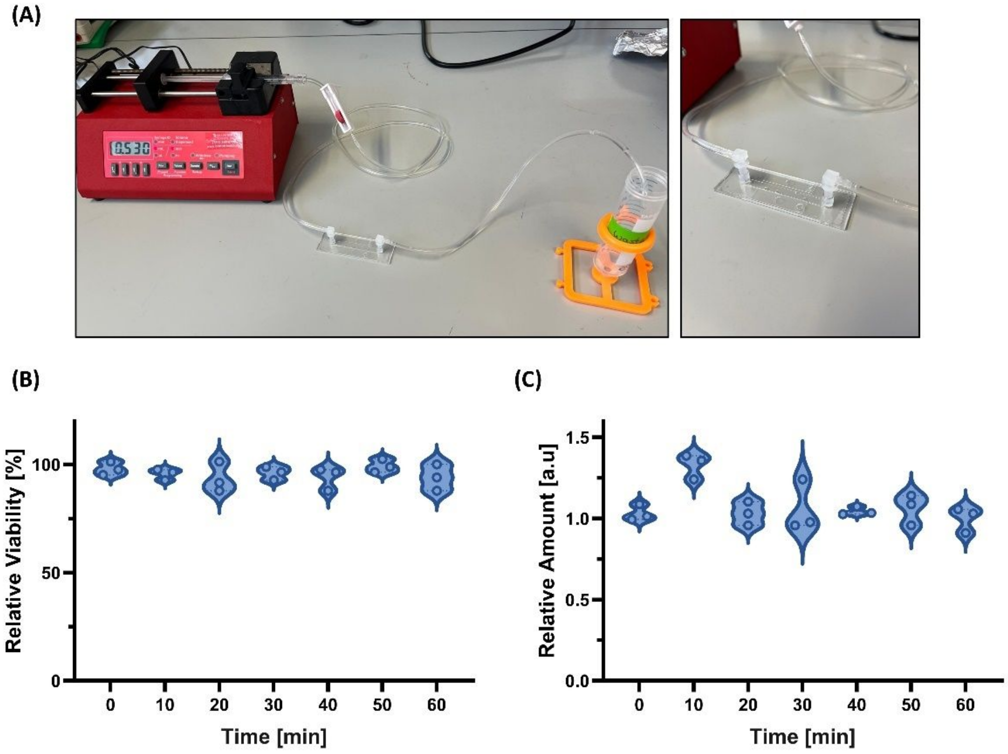

Figure S4. Experiment setup and J774 stability under flow conditions. (A) experiment setup, containing syringe pump, 1.6 mm tubes and the μ-slide I luer 0.4. **(B)** Relative Viability and **(C)** Relative amount of J774 cells measured at the endpoint of the flow path after 0, 10, 20, 30, 40, 50, and 60 minutes of under 0.53 ml/min flow rate.

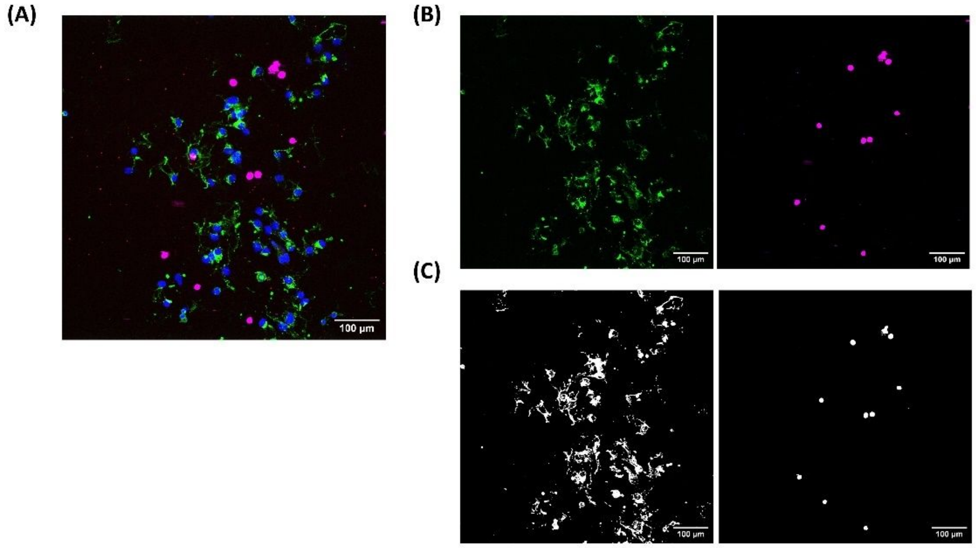

Figure S5. Macrophage to endothelial area ratio after 60 minutes of streaming and PBS washing. (A) Representative fluorescent image taken by a spinning disk confocal device of the u-slide unit under flow conditions, followed by washing with PBS. Blue = endothelial cell nuclei, Green = endothelial cell membranes, Red = nanoparticles, Purple = J774 macrophages. Scale bar = 100 µm. **(B)** Separated channel images of the green (left) and purple (right) channels, were used for calculating endothelial

and macrophage areas, respectively. Scale bar = 100 µm. **(C)** Representative images of the selected areas in the green (left) and purple (right) channels, using image analysis tools. Scale bar = 100 µm. Results are presented as mean ± SD. Statistical significance was determined using two-way ANOVA followed by Tukey's multiple comparisons test, with P ≤ 0.05 considered significant.

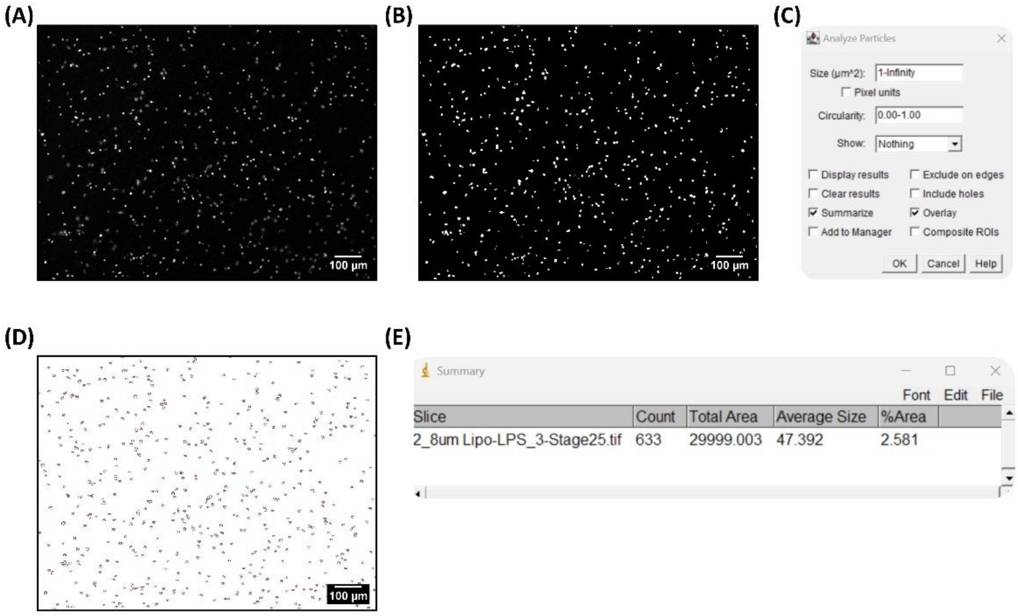

Figure S6. Transwell migration assay analysis using ImageJ particle analysis. (A) Representative image of Hoechst staining, captured with an Inverted Leica DMI8 microscope. Scale bar = 100 µm. **(B)** Processed binary image. Scale bar = 100 µm. **(C)** Application of the built-in particle analysis algorithm, with size set to 1-infinity µm² and circularity set to 0-1. **(D)** Overlay image showing the borders of the identified particles. Scale bar = 100 µm. **(E)** The measurements include the number of cells detected and their total area.

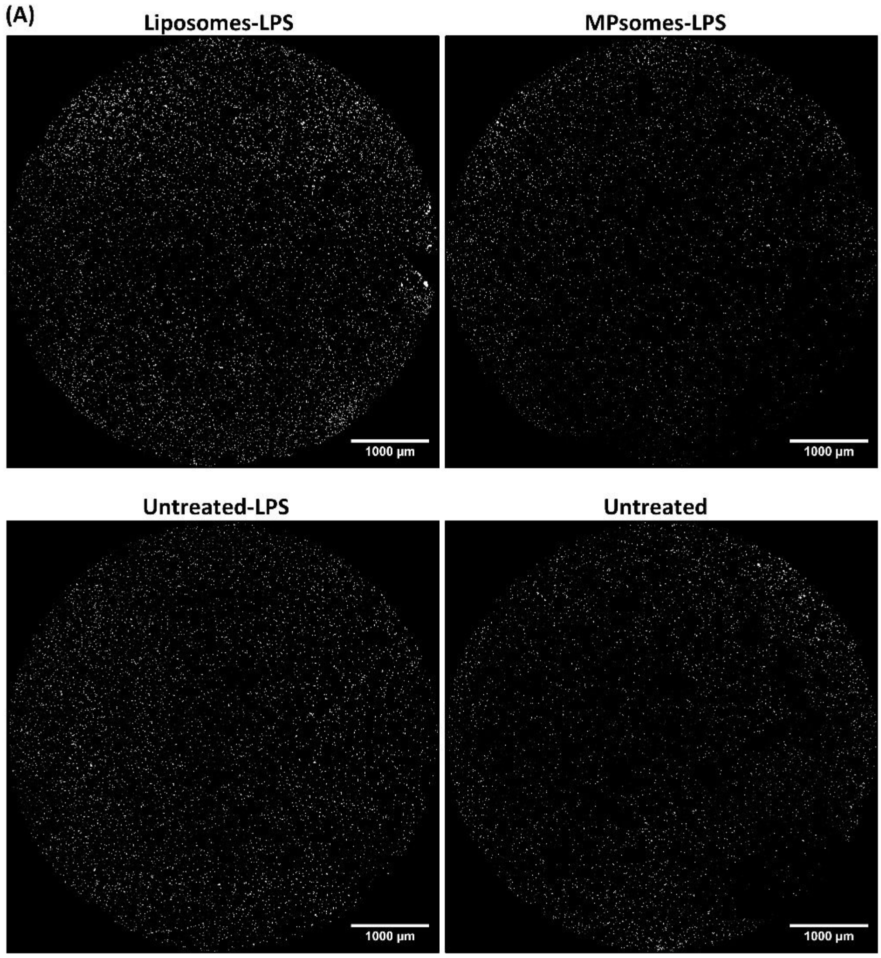

Figure S7. Area of migrating macrophages within the region of interest. (A) Representative images of the entire region of interest (5.5 mm x 5.5 mm) for each treatment group were stained with Hoechst and captured using an Inverted Leica DMI8 microscope. Scale bar = 1000 µm.

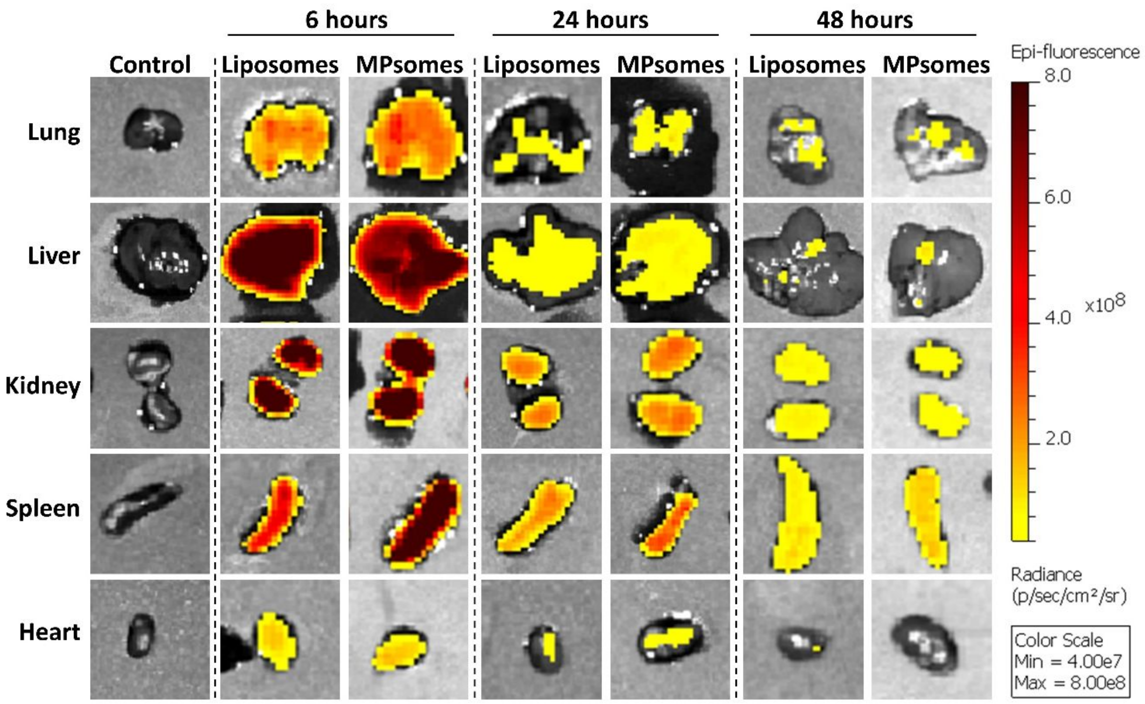

Figure S8. *Ex-vivo* IVIS imaging of Cy5-labeled NPs biodistribution. Representative *in-vivo* fluorescence (IVIS) images of major organs (lung, liver, kidney, spleen, and heart) collected from mice treated with Cy5-labeled liposomes or MPsomes at 6, 24, and 48 h post intravenous injection.

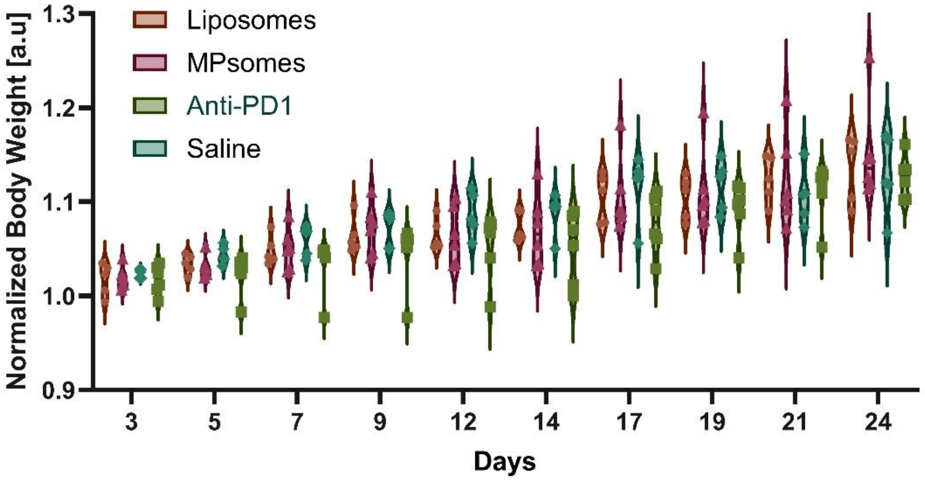

Figure S9. Normalized body weight of mice over time. Body weight of mice was monitored and normalized to their initial weight on day 0. All treatment groups maintained stable body weight, indicating no apparent systemic toxicity during the experimental period.

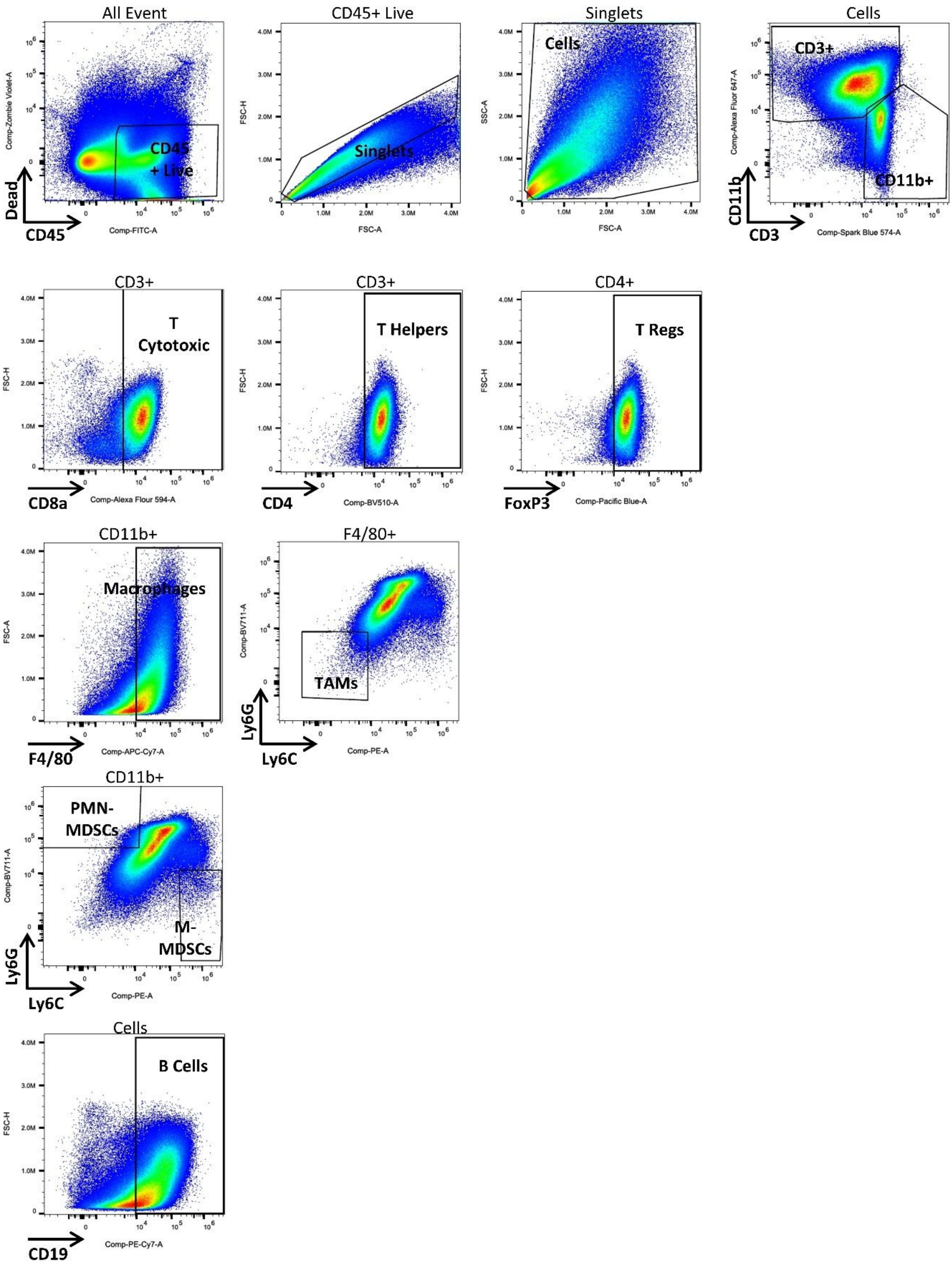

Figure S10. Gating strategy. Macrophages were identified as Zombie−, CD45+, CD11b+, F4/80+, and TAMs were defined as Zombie−, CD45+, CD11b+, F4/80+, Ly6C−, and Ly6G−; monocytic myeloid-derived suppressor cells (M-MSDCs) as Zombie−, CD45+, CD11b+, Ly6Chigh; polymorphonuclear myeloid-derived suppressor cells (PMN-MDSCs) as Zombie−, CD45+, CD11b+, Ly6Ghigh; cytotoxic T cells as Zombie−, CD45+, CD3+, CD8a+; helper T cells were identified as Zombie−, CD45+, CD4+;

regulatory T cells were identified as Zombie−, CD45+, CD4+, FoxP3+; and B cells were identified as Zombie−, CD45+, CD19+.

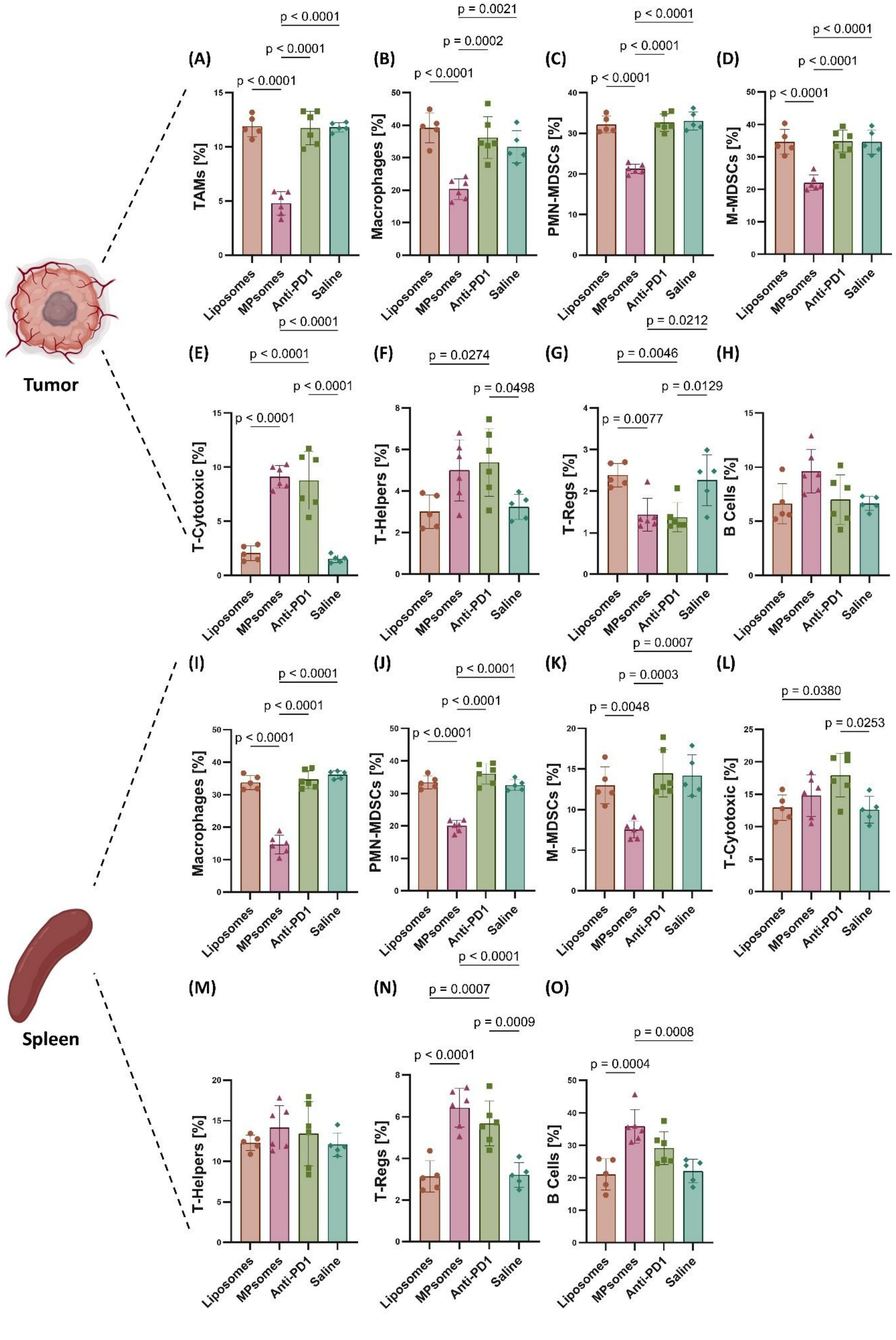

Figure S11. Immune cell population analysis in the tumor and spleen following treatment. Flow cytometry analysis showing the percentage of various immune cell subsets (of $CD45^+$ live cells) in the tumor **(A–H)** and spleen **(I–O)** after treatment with liposomes, MPsomes, anti–PD1, or saline. **(A)** TAMs, **(B)** macrophages, **(C)** PMN-MDSCs, and **(D)** M-MDSCs. **(E)** T-cytotoxic, **(F)** T-helper, **(G)** T-regulatory, and **(H)** B cells. **(I)** macrophages, **(J)** PMN-MDSCs, **(K)** M-MDSCs, **(L)** T-cytotoxic cells, **(M)** T-helper, **(N)** T-regulatory, and **(O)** B cells. Data are presented as mean ± SD; p values indicate statistical significance between groups.

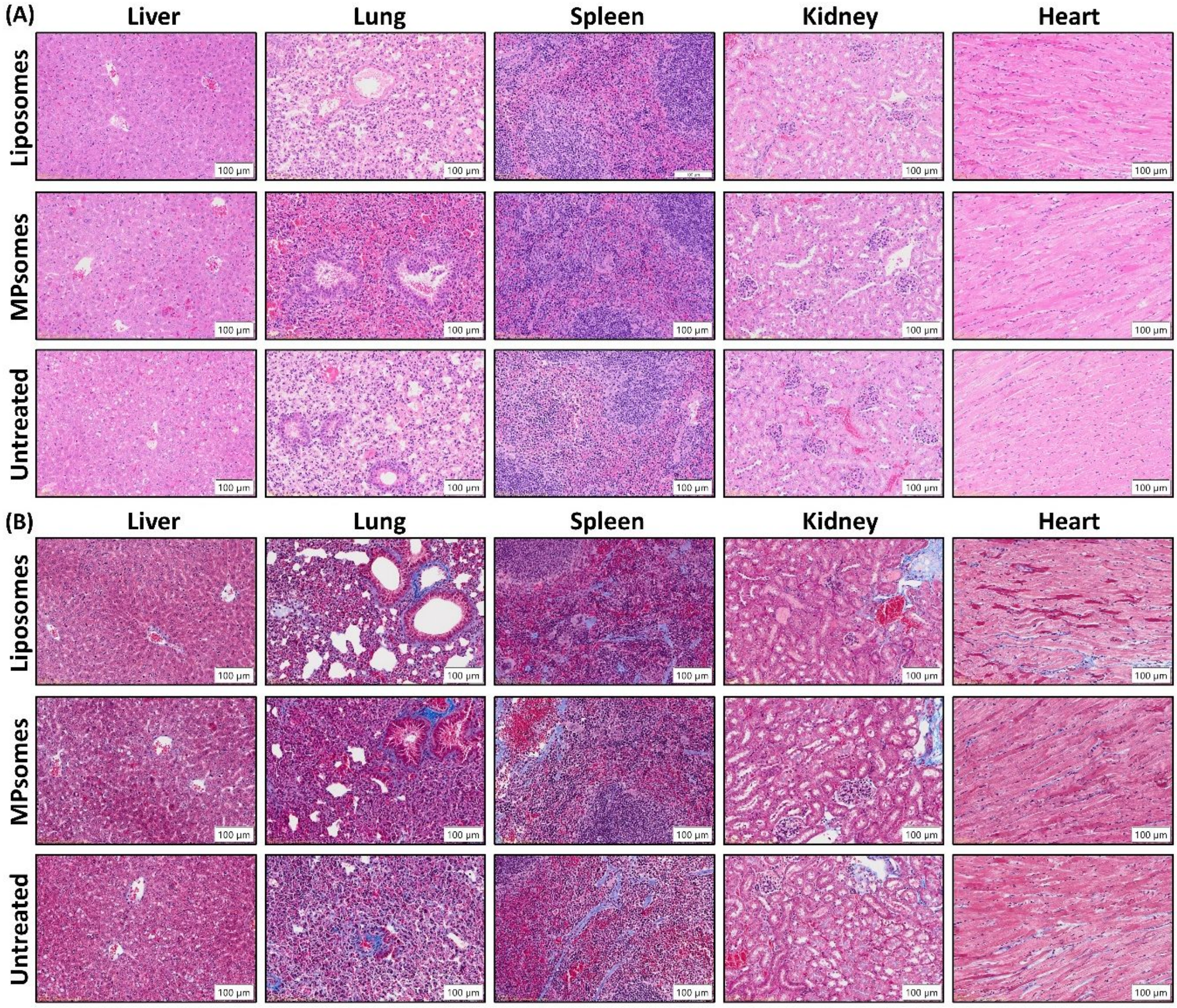

Figure S12. Histological evaluation of major organs 6 hours after nanoparticle treatment. (A) Hematoxylin and eosin (H&E) staining of liver, lung, spleen, kidney, and heart tissues collected 6 hours after intravenous administration of liposomes, MPsomes, or saline. No apparent histopathological abnormalities were observed, indicating the safety of nanoparticle treatments. **(B)** Masson's trichrome staining of the same organs showing no signs of fibrosis or tissue damage across all treatment groups. Scale bar = 100 µm.

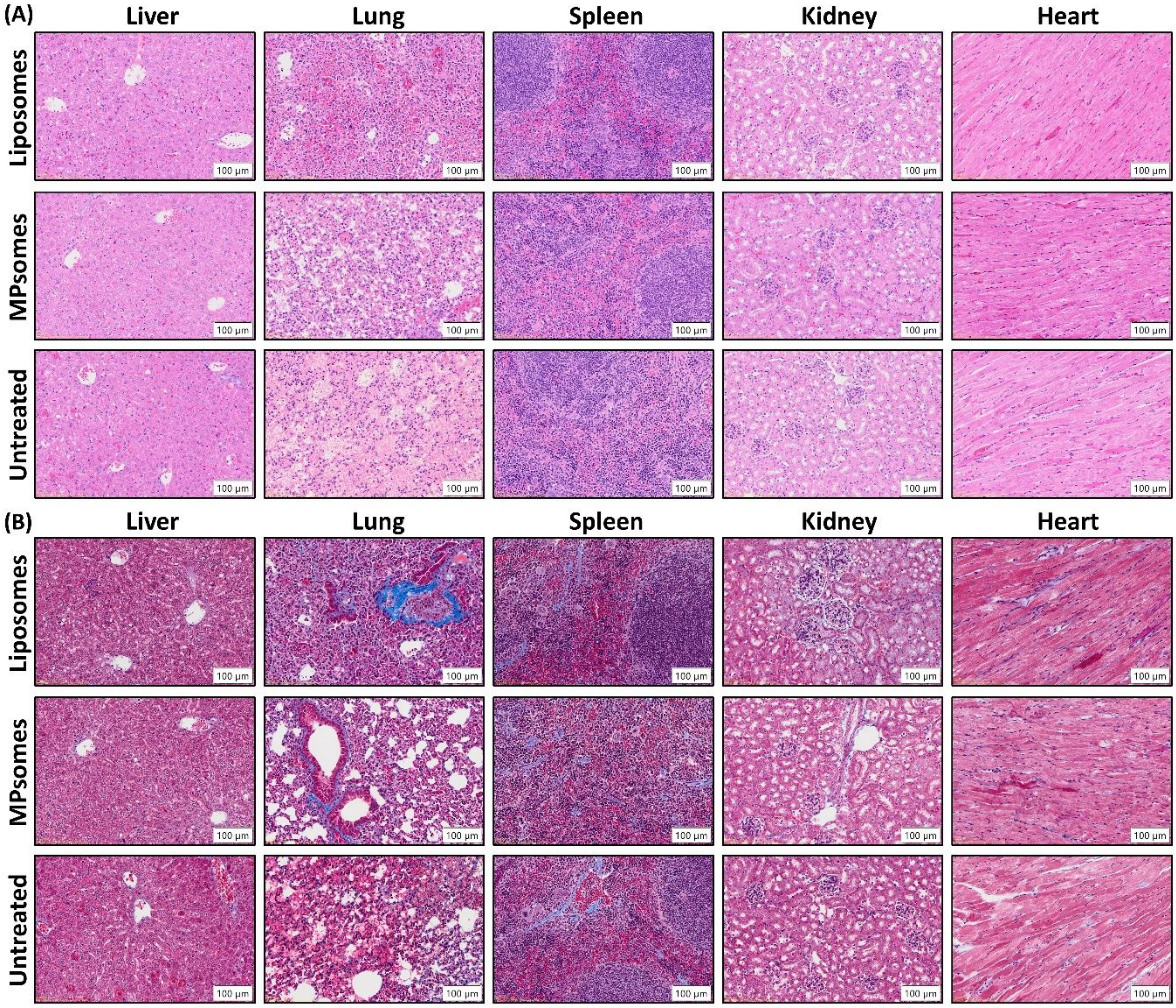

Figure S13. Histological evaluation of major organs 24 hours after nanoparticle treatment. (A) Hematoxylin and eosin (H&E) staining of liver, lung, spleen, kidney, and heart tissues collected 24 hours after intravenous administration of liposomes, MPsomes, or saline. No apparent histopathological abnormalities were observed, indicating the safety of nanoparticle treatments. **(B)** Masson's trichrome staining of the same organs showing no signs of fibrosis or tissue damage across all treatment groups. Scale bar = 100 µm.

Video S1. Streaming of macrophages and NPs on untreated or LPS-treated endothelial cells seeded on u-slide units. Fluorescent images were captured using a spinning disk confocal device under flow conditions, followed by PBS washing. Blue = endothelial cell nuclei, Green = endothelial cell membranes, Red = nanoparticles, Purple = J774 macrophages. Scale bar = 100 µm. The flow rate was set to 0.53 ml/min, with images captured every 3 minutes for a total of 60 minutes, resulting in 21 images.

Supplementary Code 1. Python script for automated DAB quantification. The following Python script was used to automatically quantify the DAB (3,3'-diaminobenzidine) signal area across all images in a given folder. The script utilizes *OpenCV*, *scikit-image*, and *pandas* libraries to separate color channels, apply color deconvolution, and compute the percentage of DAB-positive area per image.

```
import cv2
import numpy as np
import pandas as pd
import matplotlib.pyplot as plt
from skimage.color import rgb2hed
from skimage.io import imread
import os

# Folder selection
input_folder = r"C:\path"
output_csv = os.path.join(input_folder, "DAB_analysis_results.csv")

results = []

for filename in os.listdir(input_folder):
    if filename.lower().endswith(('.tif', '.jpg', '.png')):
        filepath = os.path.join(input_folder, filename)
        img = imread(filepath)

        # Convert RGB to HED (Hematoxylin-Eosin-DAB)
        hed = rgb2hed(img)
        dab_channel = hed[:, :, 2]  # DAB
        hema_channel = hed[:, :, 0]  # Hematoxylin

        # Normalize and threshold DAB
        dab_norm = (dab_channel - np.min(dab_channel)) / (np.max(dab_channel) - 
np.min(dab_channel))
        dab_thresh = dab_norm < 0.5  # DAB-positive area (brown)
        dab_percent = np.sum(dab_thresh) / dab_thresh.size * 100

        # Hematoxylin QC (image clarity / staining presence)
        hema_intensity = np.mean(hema_channel)

        results.append({
            "Image": filename,
            "DAB_positive_area_%": dab_percent,
            "Hematoxylin_intensity": hema_intensity
        })

# Save results
df = pd.DataFrame(results)
df.to_csv(output_csv, index=False)
```

```
print("Analysis completed. Results saved to:", output_csv)
```
